# Blocking Variant Surface Glycoprotein Synthesis in *Trypanosoma brucei* Triggers a General Arrest in Translation Initiation

**DOI:** 10.1371/journal.pone.0007532

**Published:** 2009-10-26

**Authors:** Terry K. Smith, Nadina Vasileva, Eva Gluenz, Stephen Terry, Neil Portman, Susanne Kramer, Mark Carrington, Shulamit Michaeli, Keith Gull, Gloria Rudenko

**Affiliations:** 1 Centre for Biomolecular Sciences, University of St. Andrews, Fife, Scotland, United Kingdom; 2 The Peter Medawar Building for Pathogen Research, University of Oxford, Oxford, United Kingdom; 3 Sir William Dunn School of Pathology, University of Oxford, Oxford, United Kingdom; 4 Department of Biochemistry, University of Cambridge, Cambridge, United Kingdom; 5 Faculty of Life Sciences, Bar-Ilan University, Ramat-Gan, Israel; The Rockefeller University, United States of America

## Abstract

**Background:**

The African trypanosome *Trypanosoma brucei* is covered with a dense layer of Variant Surface Glycoprotein (VSG), which protects it from lysis by host complement via the alternative pathway in the mammalian bloodstream. Blocking VSG synthesis by the induction of *VSG* RNAi triggers an unusually precise precytokinesis cell-cycle arrest.

**Methodology/Principal Findings:**

Here, we characterise the cells arrested after the induction of *VSG* RNAi. We were able to rescue the *VSG221* RNAi induced cell-cycle arrest through expression of a second different *VSG* (*VSG117* which is not recognised by the *VSG221* RNAi) from the *VSG221* expression site. Metabolic labeling of the arrested cells showed that blocking VSG synthesis triggered a global translation arrest, with total protein synthesis reduced to less than 1–4% normal levels within 24 hours of induction of *VSG* RNAi. Analysis by electron microscopy showed that the translation arrest was coupled with rapid disassociation of ribosomes from the endoplasmic reticulum. Polysome analysis showed a drastic decrease in polysomes in the arrested cells. No major changes were found in levels of transcription, total RNA transcript levels or global amino acid concentrations in the arrested cells.

**Conclusions:**

The cell-cycle arrest phenotype triggered by the induction of *VSG221* RNAi is not caused by siRNA toxicity, as this arrest can be alleviated if a second different *VSG* is inserted downstream of the active *VSG221* expression site promoter. Analysis of polysomes in the stalled cells showed that the translation arrest is mediated at the level of translation initiation rather than elongation. The cell-cycle arrest induced in the presence of a VSG synthesis block is reversible, suggesting that VSG synthesis and/or trafficking to the cell surface could be monitored during the cell-cycle as part of a specific cell-cycle checkpoint.

## Introduction

African trypanosomes are masters of extracellular survival in the mammalian bloodstream, where they multiply in the face of continuous host immune attack both from antibodies and the complement system. Of critical importance for the bloodstream form trypanosome is a dense protective layer of Variant Surface Glycoprotein (VSG), which shields invariant surface receptors from recognition [1]. Eventually the host mounts an effective antibody response against a given VSG variant, whereby B-cell responses against the predominant VSG play a critical role [2]. However, as new VSG switch variants continuously arise within the population, these escape recognition and form the next wave of infection. This highly sophisticated strategy of antigenic variation (reviewed in: [3], [4], [5]) allows the trypanosome to maintain a chronic infection.

An individual trypanosome encodes a vast repertoire of more than 1500 VSGs which are highly divergent in sequence [6]. In fact, it has been estimated that in *T. brucei* 927 about 60% of the VSGs are unique, with the rest occurring in very small subfamilies [7]. Despite this great dissimilarity at the sequence level, VSGs with different amino acid sequences have a highly conserved tertiary structure [8], [9]. This conservation in VSG shape presumably allows a trypanosome switching from one VSG type to another to form a protective coat composed of different VSG types. There are about 5×10^6^ VSG dimers per cell, which are attached to the cell surface through a glycosylphosphatidylinositol (GPI) anchor [10]. This makes the VSG layer on the trypanosome cell surface a very dense but highly fluid barrier. Extremely high rates of VSG endocytosis allow the trypanosome to continuously exchange the VSG on its surface [11]. Trypanosome motility coupled with these high rates of endocytosis allow the trypanosome to rapidly remove VSG-antibody complexes, providing protection from low titres of anti-VSG antibodies [12].

We have shown previously that VSG is essential in bloodstream form *T. brucei*, even *in vitro* in the absence of antibodies or complement. Blocking VSG synthesis results in a very striking and precise precytokinesis cell-cycle arrest with no re-initiation of S phase [13]. The precision of this cell-cycle arrest argues that VSG synthesis is monitored as part of a cell-cycle checkpoint, whereby progression is halted in the absence of sufficient VSG [13]. The unusually tight nature of this precytokinesis cell cycle arrest phenotype is unique in bloodstream form *T. brucei*, as other bloodstream form RNAi cytokinesis mutants described thus far have phenotypes whereby cells continue to attempt cytokinesis while subsequently re-entering S-phase in a new cell-cycle [14], [15], [16], [17], [18], [19]. For example, depletion of the *GPI8* catalytic subunit of the GPI: protein transamidase complex in bloodstream form *T. brucei* (resulting in the accumulation of unanchored VSG) results in a precytokinesis arrest [19]. Alternatively, inhibition of synthesis of a variety of flagellar proteins in bloodstream form *T. brucei* including the basal body and flagellar protein KMP-11 [16], [18] or aurora kinase-1 (TbAUK1) and related proteins results in an inhibition of cytokinesis [14], [15]. However these different precytokinesis cell-cycle arrest phenotypes are all imprecise, as the arrested cells repeatedly re-enter S-phase, and show characteristic multinuclear, multikinetoplast and multiflagellar phenotypes.

In contrast, cells in which *VSG221* RNAi has been induced are precisely stalled precytokinesis with two nuclei and two kinetoplasts, and show no indication of cleavage furrow initiation or re-entry into S-phase. The fact that the arrested cells induced by *VSG* RNAi do not re-enter S-phase, and the precision of the precytokinesis block suggest that VSG synthesis or transport could be sensed through a mechanism that interacts with the trypanosome cell-cycle. It is likely that in the absence of VSG synthesis or transport to the cell surface, a checkpoint is activated which accurately stops cell-cycle progression, preventing further cell growth and an increase in cell volume, which would cause a dilution of the cell surface VSG.

Here we demonstrate that the precise precytokinesis arrest triggered by the induction of *VSG* RNAi, is due to a block in VSG synthesis rather than toxic effects caused by large amounts of siRNAs derived from the ablated *VSG* transcript. We show that the *VSG* RNAi induced cell-cycle arrest could be rescued if a second different *VSG*, which is not recognised by the *VSG* RNAi, was introduced into the same *VSG* expression site. Strikingly, we show that blocking VSG synthesis triggered a global down-regulation of protein synthesis down to less than 1–4% normal levels. This translation arrest was correlated with disassociation of ribosomes from the endoplasmic reticulum (ER) and a drastic reduction in polysomes, arguing that the translation arrest was operating at the level of translation initiation. Additionally, we show that the precise precytokinesis cell-cycle arrest observed was reversible, suggesting that VSG synthesis or transport to the cell surface could be monitored as part of a cell-cycle checkpoint.

## Results

### The cell-cycle arrest triggered by *VSG221* RNAi could be rescued by expression of VSG117

**Figure 1:**
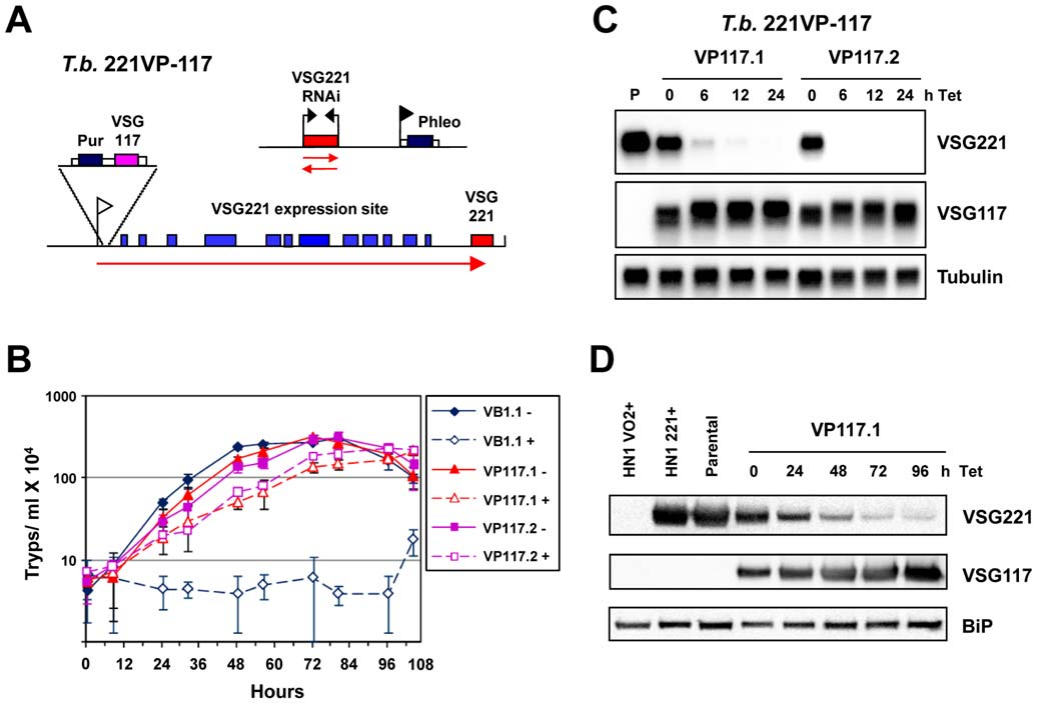
*T. brucei* expressing both VSG117 and VSG221 from the active *VSG221* expression site escapes *VSG221* RNAi induced cell-cycle arrest. A) Schematic of the *T. brucei* 221VP-117 cell line. A construct containing a *VSG117* gene linked to a puromycin resistance gene (Pur) and flanked by tubulin and *VSG221* RNA processing signals (white boxes) is inserted immediately downstream of the promoter (white flag) of the active *VSG221* expression site. Various expression site associated genes within the *VSG221* expression site are indicated with blue boxes and the telomeric *VSG221* gene with a red box. This cell line also contains a *VSG221* RNAi construct linked to a phleomycin resistance gene (dark blue box) driven by an rDNA promoter (black flag). In the presence of tetracycline, transcription of a *VSG221* fragment (red box) from opposing tetracycline inducible T7 promoters (black arrows) results in *VSG221* RNAi. Relevant transcription is indicated with red arrows. B) *T. brucei* expressing both VSG117 and VSG221 from the active *VSG221* expression site escapes growth arrest in the presence of *VSG221* RNAi. The parental *T. brucei* VB1.1 cell line (VB1) expresses only VSG221 from the active *VSG221* expression site and was incubated in the presence (+) or absence (−) of tetracycline to induce *VSG221* RNAi. The *T. brucei* 221VP-117 clones VP117.1 and VP117.2 were also grown in the presence or absence of tetracycline in order to induce *VSG221* RNAi. The standard deviation of triplicate counts is indicated with error bars. C) Northern blot analysis of *T. brucei* 221VP-117 cell lines in the presence of *VSG221* RNAi. RNA from the parental (P) *T. brucei* 221VB1.1 cell line was compared with RNA from the *T. brucei* 221VP-117 clones VP117.1 and VP117.2 which had *VSG221* RNAi induced with tetracycline (Tet) for the time in hours (h) indicated above. The blots were probed for *VSG221*, *VSG117* or tubulin as a loading control. D) Western blot analysis of the *T. brucei* 221VP-117 cell line VP117.1 after the induction of *VSG221* RNAi for the time in hours indicated above. Protein lysates from *T. brucei* HN1(VO2+) expressing VSGVO2, *T. brucei* HN1(221+) expressing VSG221 or the parental *T. brucei* 221VB1.1 cell line expressing VSG221 were compared with lysates from *T. brucei* 221VP-117 clone VP117.1 in the presence of *VSG221* RNAi induced for the time in hours indicated above. The Western blot was probed with antibody against VSG221, VSG117 or BiP as a loading control.

Blocking VSG synthesis by inducing *VSG* RNAi results in a very striking and specific cell-cycle arrest whereby cells stall prior to cytokinesis without reinitiating S-phase [13]. However, a concern with this experimental approach, is that the induction of *VSG* RNAi could result in high levels of siRNA generated from the RNAi mediated degradation of the highly abundant *VSG* transcript, which is efficiently ablated down to 1–2% normal levels [13]. In order to determine if toxic effects of the *VSG221* siRNA were causing the growth arrest, we generated trypanosomes expressing two different VSGs from the same *VSG* expression site (Fig. 1A). The parental *T. brucei* 221VB1.1 line expresses a telomeric *VSG221* gene, and contains a construct allowing the induction of *VSG221* RNAi. These cells cease growth very rapidly in the presence of tetracycline induced *VSG221* RNAi (Fig. 1B) [13]. In this cell line, a construct containing a *VSG117* gene was inserted immediately downstream of the promoter of the *VSG221* expression site to produce the *T. brucei* 221VP117 cell line (Fig. 1A).

Two independent *T. brucei* 221VP117 clones were generated which express two *VSG*s from the same *VSG* expression site. Approximately equal amounts of both VSG117 and VSG221 transcript and protein (Fig. 1C and 1D) were produced in these cells, and both VSGs were present on the cell surface (result not shown). Although these two *VSG* genes were expressed from the same *VSG* expression site, the amount of transcript and protein produced from each *VSG* appeared to be about half normal levels (Fig. 1C and 1D). When *VSG221* transcript was knocked down by the induction of *VSG221* RNAi, both *VSG117* transcript and protein increased. This indicates that the maximum amount of *VSG* transcript that can be expressed is restricted, possibly due to either a limiting *VSG* transcript stabilising protein or a restricting aspect of the RNA processing machinery. Double-expressing trypanosomes had been thought to express more VSG than normal, and it had been speculated that these trypanosomes expressing two different VSGs on their surface could have a larger volume than normal [20]. However, in agreement with our finding that “double-expressor” trypanosomes expressing both VSG117 and VSG221 on their surface had levels of total VSG comparable to parental trypanosomes expressing just VSG221, we did not find that the “double-expressors” had a significantly larger cell volume (Fig. S1).

As *VSG117* and *VSG221* are dissimilar in sequence, *VSG117* transcript is not targeted by *VSG221* RNAi. As expected, after the induction of *VSG221* RNAi with tetracycline for 6 hours, the *VSG221* transcript had almost completely disappeared, while levels of the *VSG117* transcript did not decrease (Fig. 1C). Disappearance of the *VSG221* transcript was followed by decrease of VSG221 protein from the cells where *VSG221* RNAi had been induced (Fig. 1D), presumably as a result of protein turnover, and dilution through cell growth. The induction of *VSG221* RNAi in the *T. brucei* 221VP117 cells did not lead to significant growth arrest, showing that the VSG117 protein can compensate for lack of VSG221 (Fig. 1B). These results demonstrate that the drastic growth arrest induced in the parental *T. brucei* 221VB1.1 cells in the presence of *VSG221* RNAi was not a consequence of siRNA toxicity, as *VSG221* siRNA was also being produced in the rescued *T. brucei* 221VP117 lines in the presence of tetracycline. In contrast, these results argue that the precise precytokinesis arrest observed after the induction of *VSG* RNAi is a direct consequence of blocking VSG synthesis. Attempts to establish if high level expression of a different surface protein on the surface of bloodstream form *T. brucei* could complement for lack of VSG synthesis were unsuccessful. We inserted a copy of a procyclin gene in the active *VSG221* expression site, however high level expression of procyclin appeared to be toxic in bloodstream form *T. brucei* (S.T. and G.R. unpublished results).

### Blocking VSG synthesis triggers a global translation arrest

**Figure 2:**
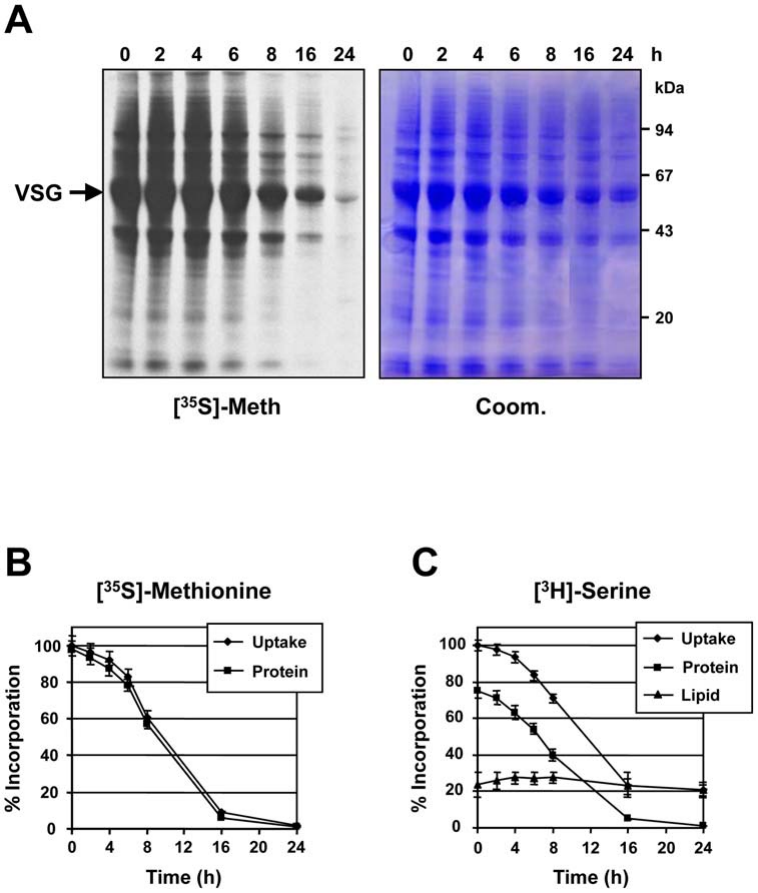
Blocking VSG synthesis triggers a global translation arrest in *T. brucei*. A) *T. brucei* 221VG1.1 cells had VSG synthesis blocked by the induction of *VSG221* RNAi for the time indicated in hours (h) prior to labeling with [^35^S]-methionine for one hour. Proteins were separated on an SDS-PAGE gel. The left panel shows [^35^S]-labeled proteins detected by fluorography ([^35^S]-Meth.). On the right is the corresponding Coomassie stained gel (Coom.). Protein sizes are indicated in kiloDaltons (kDa). B) Triplicate samples of the [^35^S]-methionine labeled cells were processed to determine the mean rate) of [^35^S]-methionine uptake and incorporation into total protein after the induction of *VSG221* RNAi for the time indicated in hours (h) with the standard deviation indicated with error bars. C) *VSG221* RNAi was induced in *T. brucei* 221VG1.1 cells for the time indicated in hours (h) prior to labeling for one hour with [^3^H]-serine. Replicate aliquots of the labeled cells were processed, and incorporation of [^3^H]-serine radiolabel into either the whole cell (uptake), total protein or lipid fractions was determined. The values show the means and standard deviations (indicated with error bars) of three separate labeling experiments, whereby the values at time 0 are normalised to 100%.

As blocking VSG synthesis triggers a cell-cycle arrest and a block in cell growth, we investigated if further metabolic activities were compromised. Total protein synthesis was followed by metabolic labeling of *T. brucei* 221VG1.1 cells with [^35^S]-methionine where *VSG* RNAi was induced for different times over a 24 hour period. Surprisingly, total protein synthesis decreased dramatically in a time dependent manner, and was reduced to less than 1% after 24 hours (Fig. 2A and 2B). In contrast, the total amount of protein as detected using Coomassie stained gels remained largely unchanged (Fig. 2A). As incorporation of [^35^S]-methionine into protein decreased, uptake also decreased, consistent with the cell maintaining a normal intracellular level of the amino acid even under conditions where protein synthesis was not draining the intracellular pool (Fig. 2B). In parallel, we investigated [^3^H]-serine uptake and incorporation into protein and lipid (Fig. 2C, Fig. S2). After the induction of a VSG synthesis block, the decrease in incorporation of [^3^H]-serine into protein resembled that seen monitoring incorporation of [^35^S]-methionine. However, incorporation of [^3^H]-serine into the lipid fraction remained unchanged over 24 hours. Using a variety of approaches, we have found that lipid synthesis was unimpaired in *T. brucei* 221VG1.1 stalled by the induction of *VSG221* RNAi for up to 24 hours (T. K. S. unpublished results). As a consequence, the uptake of [^3^H]-serine, while decreasing as a consequence of the translation arrest, reached equilibrium at about 20% of its original level. This remaining serine uptake supplies the unchanged serine utilisation in the formation of serine containing phospholipids i.e. phosphatidylserine (T. K. S. unpublished results).

One mechanism for mediating a translation block is by a perturbation of amino acid transport resulting in a reduction in intracellular amino acid concentrations. We therefore measured amino acid pools in uninduced *T. brucei* 221VG1.1 cells compared with cells where V*SG221* RNAi had been induced for 24 hours (Table 1). No drastic reductions in amino acid levels were observed with the exception of levels of serine and glutamine, which were reduced to approximately 33% and 22% of normal levels respectively. Although this reduction is significant, it is unlikely to result in the observed extreme translation block, which is down to less than 1–4% normal levels. The intracellular concentrations of both glycine and arginine surprisingly increase upon induction of *VSG* RNAi, which may be associated with the general slow down in other specific metabolic pathways. It should also be noted that these amino acids are also used for cellular functions other than protein synthesis. Serine is used for phospholipid biosynthesis and glutamine as a donor for amino-transferases while glycine may be used in the glycine cleavage system and arginine is utilised in polyamine *de novo* synthesis.

**Table 1:** Intracellular amino acid concentrations in *T. brucei* in the presence (+) or absence (−) of tetracycline induced *VSG* RNAi for 24 hours.

Intracellular amino acid concentration (µM)^a^
Amino Acid	VSG RNAi (−)	VSG RNAi (+)	% of wild type
Asp	113.5±5.8	86.3±4.9	76
Glu	243.1±8.5	169.0±6.4	70
Gly	306.2±9.3	545.6±13.8	178
Ala	68.9±3.6	61.8±4.6	90
Val	158.8±4.5	135.1±4.0	85
Ileu	207.8±6.9	174.1±4.6	84
Leu	145.6±4.5	126.7±3.8	87
Gln	841.3±7.6	184.1±4.5	22^b^
Ser	94.4±3.0	31.1±1.3	33^b^
Thr	45.9±2.2	33.3±1.8	73
Arg	69.9±3.5	112.3±9.4	161
Lys	55.6±7.8	53.7±6.2	97
Tyr	82.6±5.6	72.4±5.6	88
Phe	27.0±1.9	21.4±2.2	79
Try	4.7±0.4	5.9±0.7	126
Meth	379.8±8.7	354.8±6.7	93
Pro	79.7±3.4	79.3±4.1	100

a Amino acid concentrations within the cells were determined as described in Materials and Methods. The intracellular concentration was calculated using the cell volume 5.89 µl/10^8^ cells [59]. Values are means±SD of three separate determinations normalised using the internal standard norleucine.

b Significant difference (*P*<0.05).

We wanted to investigate if the observed translation arrest was a downstream nonspecific effect of a lethal RNAi phenotype. We therefore additionally performed [^35^S]-methionine metabolic labeling experiments in bloodstream form *T. brucei* stalled after the induction of RNAi against clathrin [21], the flagellar protein PFR2 [18], tubulin [22] and actin [23] (Fig. S3). RNAi mediated inhibition of these different proteins in bloodstream form *T. brucei* results in inhibition of growth within 24 hours, with eventual cell death. Inhibition of tubulin and PFR2 synthesis results in an imprecise precytokinesis arrest and cells with multiple nuclei and multiple flagella [18], [22]. Knock-down of clathrin and actin interferes with vesicular trafficking, producing an enlarged flagellar pocket or “big-eye” phenotype and rapid cell death [21], [23] [24].

Although inhibition of tubulin synthesis for 24 hours quickly arrests cells which later die, there was no evidence for significantly reduced translation in these stalled cells (Fig. S3). Inhibition of PFR2 synthesis resulted in translation reduced to about 40% normal levels, which is still at least ten to forty fold higher than that observed after blocking VSG synthesis. Inhibition of clathrin synthesis resulted in a reduction in translation to about 19% normal levels, and blocking actin synthesis resulted in reduction in levels of translation to about 9% normal. However, knock-down of these last two proteins would also be expected to interfere with endocytosis, and therefore with VSG recycling [12], [21], [23], [24]. It is therefore unclear if the observed translation arrest in these cells was a consequence of a disruption of deposition of VSG on the cell surface. The fact that RNAi mediated knock-down of tubulin effectively stalls cell growth without a significant decrease in translation, indicates that reduced protein synthesis is not always a consequence of a lethal RNAi phenotype. In addition, there are a number of examples of conditional knock-outs of metabolic enzymes generating a lethal phenotype prior to disruptions to translation associated with a dying cell. These include conditional knock-outs of phosphatidylinositol synthase [25], *myo*-inositol-3-phosphate synthase [26], and cytidyl ethanolamine-phosphate transferase [27].

### RNA metabolism in cells stalled by the induction of *VSG221* RNAi

**Figure 3:**
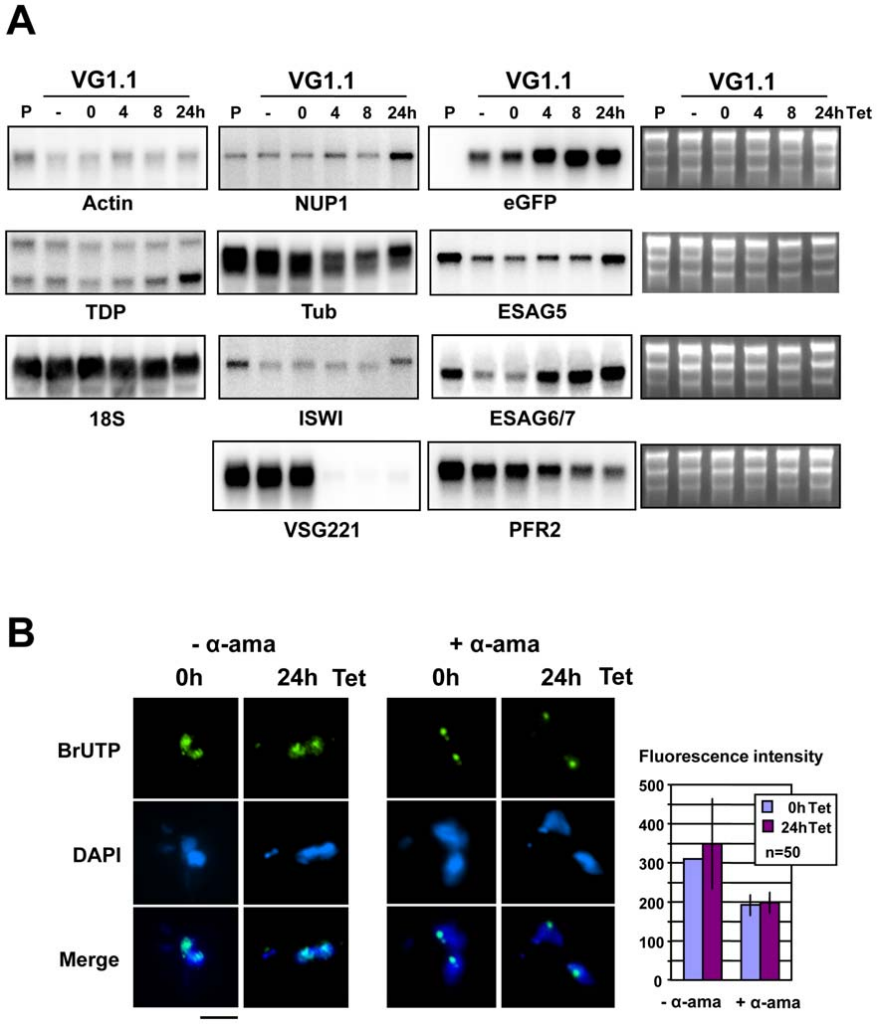
Transcription analysis of cells where VSG synthesis is blocked. A) Northern blot analysis of *T. brucei* 221VG1.1 cells where *VSG221* RNAi had been induced for the time indicated above in hours (h). The parental (P) *T. brucei* 90-13 cell line does not contain the *VSG221* RNAi construct. The *T. brucei* 221VG1.1 cell line was either not incubated with tetracycline (− Tet) or had *VSG221* RNAi induced with tetracycline for the times indicated above. Blots were hybridised with probes for Actin, *NUP1*, *eGFP* (present in the active *VSG221* ES), *TDP1*, tubulin (Tub), *ESAG5*, 18S rRNA, *ISWI*, *ESAG6/7, VSG221* and *PFR2*. Ethidium stains of the gels are indicated on the right to indicate total RNA loaded. B) Transcription analysis of *T. brucei* 221VG1.1 where VSG synthesis was blocked by the induction of *VSG221* RNAi with tetracycline (Tet) for 0 or 24 hours (h). Cells were incubated with BrUTP to label nascent transcripts and subsequently incubated with an anti-BrdUTP antibody, and a secondary antibody coupled to Alexa 488. DNA was stained with DAPI. A normal precytokinesis cell (0 h) is compared with a precytokinesis cell arising after the induction of *VSG* RNAi for 24 hours (24 h). The experiment was performed in the absence of α-amanitin (− α-ama) to visualise total transcription, or in the presence of 200 µg ml^−1^ α-amanitin to inhibit transcription by RNA polymerases II and III and visualise transcription by RNA polymerase I (+ α-ama). The scale bar indicates 4 µm. Quantitation of transcription as fluorescence in the FITC channel is in arbitrary units using 50 cells per time point, with standard deviation indicated with error bars.

We next investigated if this total block in protein synthesis was caused by changes in RNA transcript stabilities or a global transcription arrest in the stalled *T. brucei* VG1.1 cells. Northern blot analysis of *T. brucei* 221VG1.1 [13] showed rapid ablation of *VSG221* transcript within 4 hours of induction of *VSG221* RNAi (Fig. 3A). The blots were subsequently hybridised with probes for a range of housekeeping genes including the chromatin proteins *TDP1* [28] and *TbISWI* [29], *NUP1* [30], structural proteins like the flagellar protein *PFR2* [31], actin, tubulin and the 18S ribosomal RNA as well as *eGFP* (which is present in the active *VSG221* expression site [13]), *ESAG5* and *ESAG6/7*. We did not observe any drastic decreases in transcript levels, certainly not down to levels that would explain the observed global translation arrest (Fig. 3A, Fig. S4). The minor decrease in PFR2 and tubulin transcript after the induction of *VSG* RNAi could be a consequence of feedback mechanisms down-regulating transcripts in cells that are stalled in the cell-cycle and therefore not in need of these structural proteins. In addition, we found no evidence that induction of *VSG221* RNAi resulted in a decrease in the level of transcripts derived from the active *VSG221* expression site.

Recently, a novel stress response has been described in *T. brucei* where RNAi mediated silencing of the signal-recognition particle (SRP) receptor results in silencing of spliced leader RNA transcription and a significant reduction in total mRNA [32]. However, no reduction in spliced leader RNA was found in *T. brucei* 221VG1.1 cells after the induction of *VSG221* RNAi for up to 24 hours (Fig. S5). Therefore the translation arrest observed after the induction of *VSG221* RNAi must be operating through a different stress-response pathway than that involved in the spliced leader RNA silencing (SLS) response.

To investigate if transcription was arrested in the stalled cells, we monitored levels of total transcription in *T. brucei* 221VB1.1 by incorporating BrUTP into nascent transcripts in permeabilised cells (Fig. 3B). The incorporated BrUTP nucleotide analogue can be detected using an antibody against BrdUTP [33]. We compared levels of BrUTP incorporation in precytokinesis cells in the absence or presence of *VSG221* RNAi. After 24 hours tetracycline induction of *VSG221* RNAi, arrested cells had levels of total transcription that appeared comparable to that in untreated cells immediately prior to cytokinesis (Fig. 3B, -α-ama). The toxin α-amanitin inhibits RNA polymerase II and III transcription at concentrations of 200 µg ml^−1^, leaving transcription by RNA polymerase I unaffected. As seen in Fig. 3B, the inclusion of α-amanitin (+ α-ama) inhibited all transcription except for that of the rRNA in the nucleolus, as well as transcription of the active *VSG* expression site located in the expression site body (ESB), which is seen as a small discrete spot [34]. There was no discernible difference in transcription of RNA Pol I after blocking VSG synthesis for 24 hours. Therefore the total block in protein synthesis was not caused by a general arrest in transcription.

### Induction of a VSG synthesis block results in disassociation of ribosomes from the ER and a reduction in polysomes

**Figure 4:**
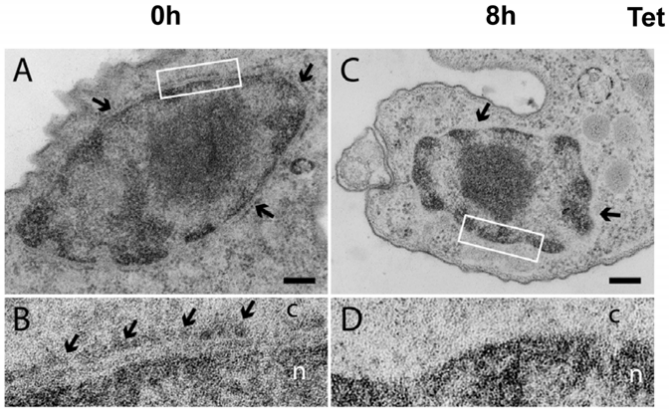
Ultrastructural evidence for disassociation of ribosomes from the endoplasmic reticulum after blocking VSG synthesis. A) In non-induced *T. brucei* 221VG1.1 cells (0 h), the outer membrane of the nuclear envelope is studded with ribosomes (arrows). Membranes are not contrasted because cells are not treated with osmium tetroxide. Scale bars represent 200 nm. B) A region of the nuclear envelope indicated with a white box in (A) shown at 4x higher magnification. An array of membrane-associated ribosomes is indicated with arrows. c = cytoplasm and n = nucleoplasm. C) After induction of *VSG* RNAi for 8 hours, large stretches of nuclear envelope are devoid of ribosomes (arrows). D) A region of the nuclear envelope indicated with a white box in (C) shown at 4x higher magnification. No membrane-associated ribosomes are observed.

If the observed translation arrest was operating at the level of translation elongation, one would expect to see unaffected distribution of ribosomes on the ER. In order to investigate this, we determined the distribution of ribosomes on the endoplasmic reticulum (ER) using transmission electron microscopy (TEM) and a nonstandard TEM fixing and staining technique. Normally ribosomes are distributed uniformly on the outer membrane of the nuclear envelope, which is contiguous with the rough ER (indicated with arrows in Fig. 4A and B). After induction of *VSG221* RNAi in *T. brucei* 221VG1.1 cells for 8 hours, the nuclear envelope appeared to be devoid of significant numbers of bound ribosomes (Fig. 4C and D). This disassociation of ribosomes from the ER coincided with the previously observed decrease in protein synthesis. This result is consistent with the observed translation arrest being a consequence of disassociation of polysomes in the stalled cells rather than a scenario whereby polysomes remain intact but translation elongation is blocked.

**Figure 5:**
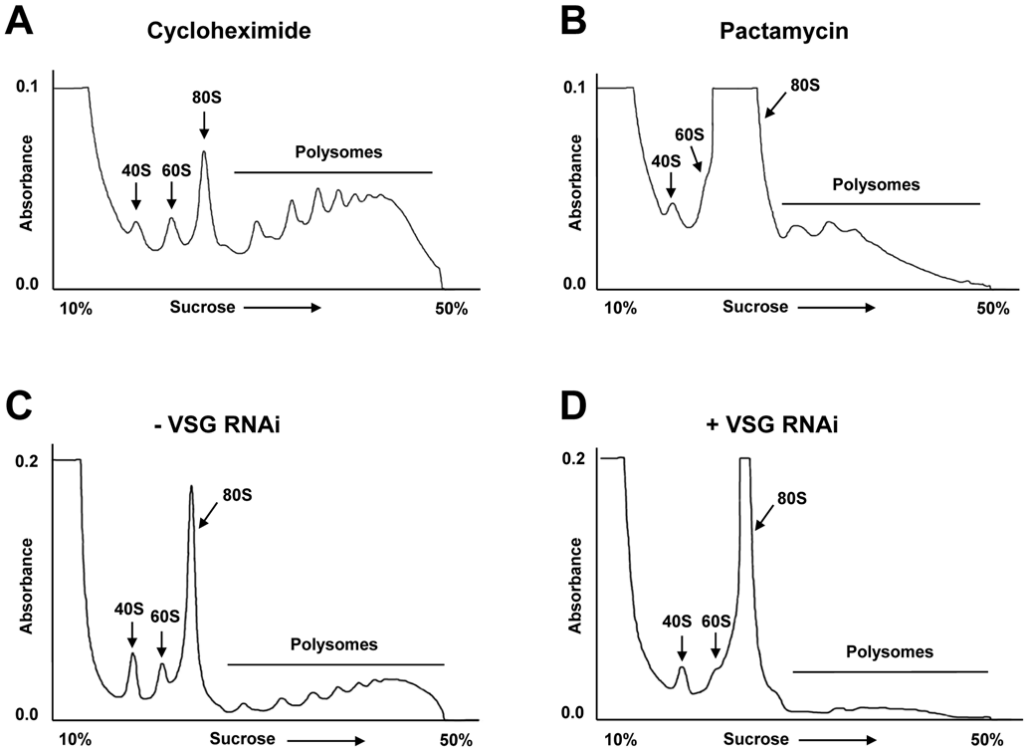
Polysome profile analysis indicates that the *VSG221* RNAi induced global translation arrest operates at the level of translation initiation. A) Polysome profile of *T. brucei* 221VG1.1 cells which have been incubated with cycloheximide (50 µg ml^−1^) to block translation elongation. The percentage of sucrose from top to bottom of the gradient is indicated on the X-axis, with the absorbance at 254 nm indicated on the Y-axis. The free ribosomes (80S) and ribosomal subunits (40S and 60S) are indicated with arrows. The polysomes (translating ribosomes bound to transcript) are indicated with a bar. B) Polysome profile of *T. brucei* 221VG1.1 cells which have been incubated with pactamycin (200 ng ml^−1^) to block translation initiation. C) Polysome profile of *T. brucei* 221VG1.1 in the absence (−) of *VSG* RNAi. D) Polysome profile of *T. brucei* 221VG1.1 where *VSG221* RNAi has been induced for 24 hours (+).

Polysomes are large complexes composed of translating ribosomes associated with mRNA transcripts which fractionate at the bottom of sucrose gradients. In contrast, free ribosomes fractionate at the top of the gradient. Polysome profiles from normal cells and cells where a *VSG* RNAi mediated block of VSG synthesis had been induced were determined by separating lysed *T. brucei* over sucrose gradients (Fig. 5). We first determined the polysome profile of *T. brucei* incubated with cycloheximide (50 µg ml^−1^), which blocks translation elongation [35]. The ribosomes as monitored by absorbance at 254 nm were primarily found in polysomes, which contain transcripts associated with the translating ribosomes (Fig. 5A). In contrast, if *T. brucei* is incubated with pactamycin (200 ng ml^−1^) which blocks translation initiation [35], [36], ribosomes disassociated from the transcripts, and there was a dramatic increase in free ribosomes (80S)(Fig. 5B).

In a similar fashion, polysome profiles were determined for *T. brucei* 221VG1.1 in the presence or absence of a VSG synthesis block. In the absence of *VSG221* RNAi (Fig. 5C), most of the ribosomes were associated with polysomes. However, after the induction of *VSG221* RNAi for 24 hours, there was a drastic decrease in polysomes and an increase in free ribosomes (80S) (Fig. 5D). These experiments demonstrate that the global translation arrest observed after the induction of *VSG221* RNAi was operating through a block in translation initiation rather than translation elongation.

We have not found evidence for striking changes in levels of a range of different *T. brucei* translation factors (eIF4A, eIF4E1-4) [37](M. Narayanan, O. Neto, M.C. and G.R. unpublished) after the induction of a *VSG* RNAi induced cell-cycle arrest. Phosphorylation of translation initiation factor eIF2α is an important potential mechanism for blocking global protein synthesis at the level of translation initiation [38], [39]. However, we have not found evidence for changes in levels or phosphorylation state of *T. brucei* eIF2α in cells stalled after the induction of *VSG* RNAi for up to 24 hours (M. Narayanan and G. R. unpublished results). Similar to what we describe here, there is a comparable reduction in polysome abundance after the induction of a heat-shock response in trypanosomes, yet no evidence for changes in the phosphorylation state of eIF2α [40]. This could indicate significant differences in translation control in trypanosomes compared with other eukaryotes.

A concern with a global translation arrest is that it is a consequence of dying cells. We know that levels of transcription, lipid synthesis and endocytosis of FITC-tomato lectin are not obviously affected in the arrested cells (Fig. 3, [13]), indicating that they are metabolically active. In order to investigate protein composition in these stalled cells, we used proteomic two dimensional Difference Gel Electrophoresis (2D-DIGE analysis) [41]. We did not see any significant up- or down-regulation of the levels of any individual protein in cells stalled after the induction of *VSG* RNAi for 8 hours, when the cell-cycle arrest is already maximal (Sup. Fig. 6). Although this type of proteomic analysis would only be expected to sample the most abundant proteins (in our case 1486 different spots), this result indicates that the arrested cells do not have a drastically different protein composition from normal. This would support the observation that the cells arrested immediately after the induction of the cell-cycle arrest are not an obviously dying system that is degrading. Because blocking VSG synthesis could be expected to result in aberrant expression of other surface proteins in the cell as a compensatory effect, we looked specifically at the procyclic specific cell surface protein procyclin. However, we did not observe that blocking the major cell surface protein (VSG) resulted in significant upregulation of procyclin, either at the RNA or the protein level (Fig. S7).

**Figure 6:**
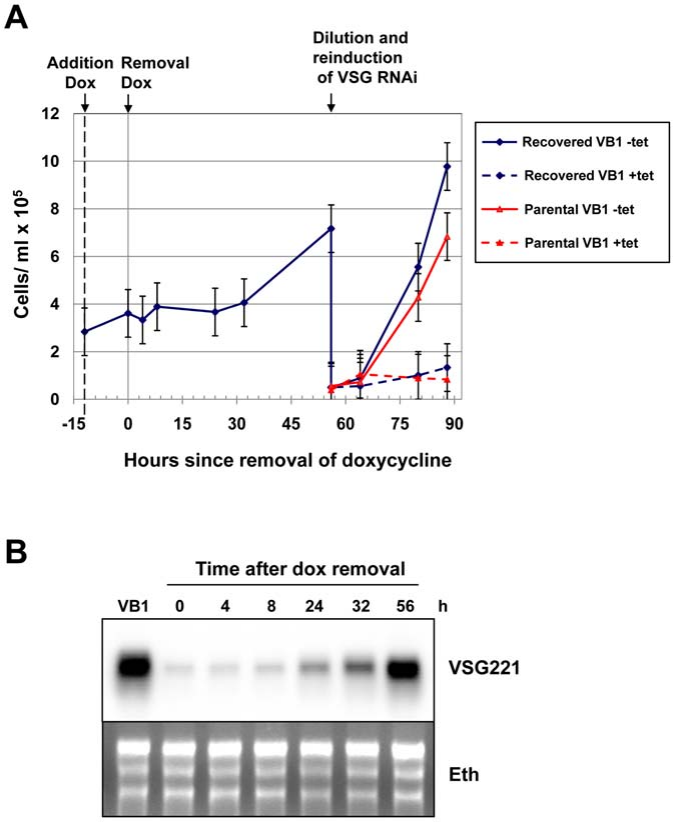
The cell-cycle arrest in *T. brucei* 221VB1.1 triggered by blocking VSG221 synthesis is reversible after the removal of doxycycline. A) Doxycycline was added to a culture of *T. brucei* 221VB1.1 (VB1) for 12 hours (indicated with an arrow labeled addition dox) to induce *VSG221* RNAi and a subsequent cell-cycle arrest. The cells were then washed to remove the doxycycline (removal dox) and growth was monitored. After an initial stalled period, the trypanosomes started to grow. In order to establish that these growing trypanosomes were still responsive to *VSG221* RNAi, these growing trypanosomes were diluted and *VSG221* RNAi was induced with 1 µg ml^−1^ tetracycline (blue dashed line). As a control, *VSG221* RNAi was also induced in the parental *T. brucei* 221VB1.1 cells (red lines). The standard deviation of counts in triplicate is indicated with error bars. B) Northern blot analysis of RNA from *T. brucei* 221VB1.1 cells following removal of doxycycline. RNA from the noninduced *T. brucei* 221VB1.1 (VB1) is compared with RNA from *T. brucei* 221VB1.1 cells where doxycycline has been removed from the cultures for the time in hours (h) indicated above. The blot was hybridised with a probe for *VSG221*. An ethidium stain (Eth) of the blot is indicated below.

### The *VSG* RNAi mediated cell-cycle arrest is reversible

As a global translation arrest is an extreme phenotype, we next determined if the cell-cycle arrest triggered by the induction of *VSG* RNAi is reversible. *T. brucei* 221VB1.1 was incubated with the water soluble tetracycline analogue doxycycline for 12 hours to induce *VSG221* RNAi and induce a cell-cycle arrest (Fig. 6A). Doxycycline was then removed by washing the cells and transferring them to fresh medium. *VSG* RNAi was induced using 300 pg ml^−1^ doxycycline, as this concentration reproducibly triggered a maximum cell cycle arrest, yet was low enough to facilitate the washing step (Fig. S8). Comparable cell cycle arrest results were obtained using 2 ng ml^−1^ tetracycline. However using tetracycline resulted in greater experimental variability compared with doxycycline, possibly due to differences in its solubility or cell permeability.

After an initial lag of about 24 hours following doxycycline removal, the cells resumed growth, presumably due to the translation arrest impeding rapid recovery. Northern blot analysis showed that *VSG221* transcript levels returned back to normal levels 56 hours after the stalled cells were removed from doxycycline (Fig. 6B). These recovered cells stalled abruptly if *VSG221* RNAi was induced again, showing that they were still fully responsive to *VSG* RNAi. To determine the cloning efficiency of this experimental procedure, serial dilutions were plated out in microtitre dishes. The observed rescued trypanosomes arose reliably in about 15% of the wells when less than 80 stalled cells were allowed to recover, which is about 30x less efficient than that of cloning the parental *T. brucei* 221VB1.1 line (Fig. S9). This relatively low cloning efficiency is presumably due to deleterious aspects of the *VSG* RNAi induction, including the generation of internalised flagella in some of the cells [13]. However, our recovered cells arise more rapidly than would be expected from generation by background mutations, as the mutation rate in *T. brucei* has been estimated to be about 10^−9^ per bp per cell generation [42]. These results argue that the cell-cycle arrest triggered by blocking VSG synthesis is reversible. After a brief arrested state, some of the cells re-enter the cell-cycle and resume dividing. The reversibility of the precise precytokinesis cell-cycle arrest suggests that this phenomenon is not necessarily a dead end pathway. It could possibly have relevance in allowing the trypanosome to react to fluctuations in either VSG synthesis and/or deposition of VSG on the cell surface that are less drastic than those described here, where essentially all VSG synthesis is blocked for a significant period of time.

## Discussion

We show that although the induction of *VSG221* RNAi normally induces a precise precytokinesis cell-cycle arrest in VSG221 expressing trypanosomes, cells did not stall in the cell-cycle if *VSG117* (which is not recognised by the *VSG221* RNAi) was also expressed from the active *VSG221* expression site. This argues that the cell-cycle arrest observed after the induction of *VSG221* RNAi is a consequence of lack of newly synthesised VSG rather than toxicity of the *VSG221* siRNA. Surprisingly, an extreme and global block in protein synthesis was induced in the stalled cells, whereby total translation was reduced to 1–4% normal levels after 24 hours induction of *VSG221* RNAi. No major changes in transcription or transcript levels were observed that explain this protein synthesis block. However, after 8 hours induction of *VSG* RNAi ribosomes appeared to have disassociated from the ER. Polysome analysis of the stalled cells showed that the translation block was operating at the level of translation initiation rather than translation elongation. Despite the striking changes in the arrested cells, particularly with regards to the global arrest in protein synthesis, the cell-cycle arrest was reversible suggesting that VSG synthesis and/or deposition on the cell-surface is possibly being monitored as part of a normal cell-cycle checkpoint.

Earlier, it has been shown that *T. brucei* can be genetically modified to express two *VSG*s from the telomere of the active *VSG* expression site [43]. We show that a second *VSG* could also be efficiently expressed if it was inserted immediately downstream of the promoter of the active *VSG* expression site rather than in its usual telomeric location. The invariably telomeric location of *VSG*s within *VSG* expression sites therefore presumably facilitates *VSG* recombinogenicity rather than being essential for expression.

Surprisingly, the precise precytokinesis arrest observed after blocking VSG synthesis coincided with an extreme and global block in protein synthesis down to less than 1–4% normal levels. No major decrease in transcription or steady state transcript levels was observed that explains this protein synthesis block. Although there was some reduction in transcripts encoding structural proteins including the flagellar protein PFR2 and tubulin, the observed reduction of just a few transcripts does not explain the global protein synthesis arrest phenotype. Similarly, Northern blots probed with spliced leader RNA specific oligonucleotides showed no drastic reduction in total mRNA (S.T. and G.R. unpublished results). These results are compatible with our observation that transcription was not detectably disrupted in these stalled cells. These data agree with previously reported results [44], which show that the *T. brucei* transcriptome remains largely unchanged in trypanosomes when confronted with a variety of stresses. This indicates that *T. brucei* does not respond to environmental challenges through transcriptional regulation, and in the absence of protein synthesis, still transcribes and then degrades large amounts of excess transcript.

In addition, there were no abrupt decreases in intracellular amino acid pools which could explain this block in protein synthesis. Incorporation of methionine and serine into protein decreased drastically. However, phospholipid synthesis was not greatly altered in these stalled cells (T.K.S. unpublished data), allowing serine uptake (at about 20% normal levels) and incorporation into phospholipids to continue. This continued uptake of serine indicates that the protein synthesis block was not a consequence of disruption in general amino acid uptake, possibly through minor perturbations in the VSG coat negatively affecting amino acid transporters. Also if this were the case, then the stalled cells would not be able to escape the cell-cycle arrest.

Polysome analysis of the stalled cells showed that the translation block was operating at the level of translation initiation rather than translation elongation. Compatible with this, after 8 hours induction of *VSG* RNAi ribosomes appeared to have disassociated from the ER. The signal sequence on nascent peptides targets the translating ribosome to the ER membrane, facilitating anchoring of the ribosome to the ER through the binding of the signal recognition particle (SRP) to SRP receptors on the ER surface [45], [46]. As VSG is the major secreted protein in bloodstream form *T. brucei*, blocking its synthesis would result in a drastic reduction in nascent peptides with signal sequences. Ribosomal subunits in mammalian cells remain membrane-bound after pharmacological inhibition of protein synthesis [45]. Even if ribosomal subunits initially remained membrane-bound upon termination of VSG translation, ongoing translation of cytosolic transcripts would result in a clearance of ribosomes from membranes [35]. This probably occurs early after induction of *VSG* RNAi, and is followed by the observed global translation arrest.

Why is there a global translation arrest in trypanosomes stalled precytokinesis after the induction of a VSG synthesis block? The first possibility is that the translation arrest is triggered as part of a general stress response, perhaps as a consequence of lack of VSG synthesis or deposition on the cell surface. In our experimental system the ER is being depleted of newly synthesised VSG, which accounts for 10% of the total protein, and which could generate a form of stress. In some eukaryotes a global translation block can be observed after the induction of the unfolded protein response (UPR) [reviewed in: [47]]. UPR is triggered by ER stress, including the accumulation of unfolded protein within the ER. If this ER stress continues for a prolonged period, translation arrests in order to stop the stream of additional protein into the overburdened ER [38], [48]. If the situation persists, the cell undergoes apoptosis [47]. Our *VSG* RNAi induced arrest does not appear to be the same as that induced by UPR, as there was no significant upregulation of the chaperone BiP [13], which is characteristic of UPR [49]. In addition, in the absence of VSG synthesis the cells stall and remain metabolically active rather than entering apoptosis. It is possible that trypanosomes have a novel stress response pathway different to UPR, which is triggered by the stress induced by a restriction operating on the deposition of VSG on the cell surface. In this regard it is striking that knock-down of actin, which would be expected to be involved in VSG recycling, also results in a striking arrest in translation (Fig. S3).

Another stress response in trypanosomes is the spliced leader RNA silencing response (SLS), which occurs after the induction of stress in the ER by pH stress, or by blocking synthesis of the signal recognition particle (SRP) receptor, which facilitates the translocation of secretory proteins across the ER [32]. The SLS response results in the down-regulation of the spliced leader RNA genes, resulting in drastic reduction in total mRNA and subsequent cell death [32]. The translation arrest observed after the induction of *VSG* RNAi is not coupled to an SLS response, as there was no reduction in spliced leader transcript after induction of *VSG* RNAi for up to 24 hours (Fig. S5). In addition, there was no significant reduction in total trans-spliced mRNA transcripts (another expected consequence of induction of an SLS response) after the induction of *VSG* RNAi for up to 24 hours (S.T. and G.R unpublished results; [32]). Therefore the ER stress caused by blocking VSG synthesis appears to use different pathways than those that trigger the spliced leader RNA silencing response in African trypanosomes.

A second possible explanation for the observed translation arrest, is that trypanosomes always transiently arrest protein synthesis when they are in the precytokinesis stage of the cell-cycle. Earlier, it has been shown in mammalian cells that protein synthesis slows down when cells are in the G2/M stage of the cell-cycle [50]. However in our case, this is unlikely to provide the explanation. After the induction of *VSG* RNAi, the stalled population of trypanosomes is up to 60% enriched for precytokinesis stage cells, but still contains cells in other stages of the cell-cycle which normally would be expected to be translationally active [13]. This scenario is not compatible with our observed block in protein synthesis down to less than 1–4% normal levels.

A third possibility is that the observed translation arrest is a general feature of RNAi phenotypes that result in growth inhibition and eventual death. This is not the case, as cells arrested as a consequence of another lethal phenotype (induction of a tubulin synthesis block) do not show reduced levels of translation (Fig. S3). In addition, we have no evidence that our cells are an obviously dying system within the 24 hour period used in these analyses. Our arrested cells are viable for relatively long periods, with virtually all cells intact and metabolically active (other than protein synthesis) after 24 hours [13]. Endocytosis of FITC-tomato lectin appears unimpaired [13], and as shown here, there was no significant drop in transcription. In addition, the proteomic analysis did not detect changes in the most abundant proteins even in fully stalled cells (Fig. S6), confirming that we are not looking at a system that is obviously falling apart. Last, arrested cells were able to reenter the cell-cycle when doxycycline was removed.

We favour an explanation whereby induction of a VSG synthesis block triggers a stress response resulting in a global translation arrest. Although it is unclear what exactly is being “sensed” in this stress response pathway, our data is most compatible with a scenario whereby the cell senses the restriction occurring from the lack of deposition of VSG on the cell surface. The fact that downregulation of actin (which is necessary for vesicular trafficking of VSG) also triggers a severe translation arrest is compatible with this possibility.

Does this arrest operate *in vivo* during an infection? Our experiments here document an extreme situation whereby virtually all VSG synthesis is rapidly blocked by the induction of *VSG* RNAi. It is unclear if anything this extreme occurs within a natural trypanosome infection. VSG synthesis also halts in “stumpy form” *T. brucei* where transcription of the active *VSG* expression site stops [51]. There are some superficial similarities between our cells arrested after the induction of a VSG synthesis block and stumpy form *T. brucei*. Similar to stumpy cells, our arrested cells are shorter and broader than normal, arguing that this morphological change could be a consequence of a restriction operating on VSG [13]. In addition, stumpy form *T. brucei* also show reduced levels of protein synthesis and a reduction in polysomes [52]. However, stumpy form *T. brucei* are stalled in G0 rather than precytokinesis, and have undergone other morphological changes that facilitate differentiation into procyclic form trypanosomes that are not seen in our stalled cells. It is unlikely that VSG synthesis is ever drastically reduced in bloodstream form *T. brucei* to the extent seen after the induction of *VSG* RNAi. However, possibly the stress response documented here has evolved to allow the trypanosome to reduce cell growth in response to fluctuations in amount of synthesised VSG rather than complete ablation of *VSG* transcript. An important feature of this stress response is reversibility, which could argue that the triggered cell-cycle arrest has biological relevance, rather than being an irreversible end-point from which the trypanosome is unable to escape. The challenge for the future will be in unravelling exactly which pathways are involved in these novel cell-cycle checkpoints.

## Materials and Methods

### Trypanosome strains, culturing and genetic modification

Bloodstream form *T. brucei* 427 was cultured in HMI-9 medium with 10% SerumPlus (SAFC Biosciences) and 10% fetal calf serum (Gibco). All trypanosomes used were bloodstream form variants derived from *T. brucei* 427 90-13 expressing VSG221 [53]. The *T. brucei* 221VB1.1 and *T. brucei* 221VG1.1 cell lines are described in [13]. The *T. brucei* 221VP117 transformants have the p221-purVSG117UTR construct inserted into the *T. brucei* 221VB1.1 cell line. The construct is integrated immediately behind the *VSG221* expression site promoter using the target fragments of the 221GP1 construct described in [54]. This construct contains the *VSG117* gene from pXS5:117VSG (gift of Jay Bangs)[55] flanked downstream by the *VSG221* UTR and polyadenylation sequences. The *VSG117* cassette is inserted downstream of the puromycin drug resistance gene, which is flanked up and downstream by α-tubulin gene RNA processing signals [54]. Homogeneity of the VSG coat expressed was ensured by maintaining trypanosome transfectants with drug markers in the active *VSG* expression site on the appropriate drug selection pressure to prevent switches to different *VSG* expression sites. Tet-system approved fetal calf serum (Clontech) was used for some experiments requiring tetracycline free conditions.

### Nucleic acid and polysome analysis

For Northern blot analysis, total RNA was purified using RNeasy RNA isolation kits (Qiagen). Approximately 1.5 µg total RNA was loaded per lane on 1.5% formaldehyde agarose gels, electrophoresed and blotted using standard protocols. Northern blots were hybridized with random primed probes radiolabeled with [^32^P]-dCTP using Amersham Ready-To-Go DNA labeling Beads (-dCTP) (GE Healthcare). Details on the exact sequence of the different Northern probes can be obtained from the authors. Blots were imaged with a Bio-Rad Personal Molecular Imager FX. Quantitation of signal was performed using Quantity One software, and radioactive signals were plotted in arbitrary units.

Polysome analysis was performed according to [36] and [40], whereby 400 ml cultures of *T. brucei* 221VG1.1 cells were grown to a density of about 7.5×10^5^ cells ml^−1^. *VSG* RNAi was induced by incubating the cells with 1 µg ml^−1^ tetracycline for 24 hours. Pactamycin treated trypanosomes were incubated with 200 ng ml^−1^ pactamycin (gift of Pfizer Global Research & Development, Groton, Connecticut, USA) for 20 minutes at 37°C, and cycloheximide-treated trypanosomes were incubated with 50 µg ml^−1^ cycloheximide for 10 minutes at 37°C prior to the start of polysome purification procedure. Cells were harvested by centrifugation at 4°C and washed with HMI-9 without serum (containing either pactamycin or cycloheximide where appropriate). Cells were resuspended in polysome buffer (120 mM KCl, 2 mM MgCl_2_, 20 mM Tris pH 7.5, 1 mM DTT) containing either cycloheximide or pactamycin where appropriate. Cells were lysed using the detergent n-octylglycoside (which does not absorb at 254 nm), loaded on top of 10%–50% sucrose gradients and centrifuged for 2 hours at 36,000 rpm at 4°C in a Beckman ultracentrifuge using a SW41 rotor. Gradients were then harvested using a peristaltic pump and analysed using an absorbance reader at 254 nm.

RNA analysis to determine the presence or absence of an SL response was performed using end-labeled 9091 oligonucleotide to detect the spliced leader and U3 oligonucleotide (1×10^5^ cpm pmol^−1^) according to [56]. After annealing at 60°C for 15 minutes, the sample was kept on ice for one minute. Next, one unit of reverse transcriptase (Expand RT, Roche Molecular Biochemicals) and one unit of RNase inhibitor (Promega) were added, and extension was performed at 42°C for 90 minutes. The reaction was analysed on a 6% polyacrylamide denaturing gel.

### Microscopy

Labeling of nascent RNA using BrUTP was performed essentially according to [34], only the Saponin (Sigma) incubation was performed at 4°C. After the transcription reaction, cells were washed and fixed in 2% paraformaldehyde and BrUTP labeled transcripts were detected with a monoclonal anti-BrdUTP antibody (Roche). For transmission electron microscopy analysis of membrane bound ribosomes, cells were fixed by addition of 2.5% glutaraldehyde directly to the culture medium followed by fixation in 2.5% glutaraldehyde in 0.1 M phosphate buffer pH 7.0 for 2 hours at room temperature without osmium tetroxide post-fixation. Cells were dehydrated in a graded series of ethanol and embedded in Epon resin. Thin sections were stained 5 minutes with 5% aqueous uranyl acetate and 1 minute with 0.1% lead citrate and examined in a FEI Tecnai 12 transmission electron microscope.

### Cell-cycle arrest reversibility and cell volume measurements

*T. brucei* 221VB1.1 was cultured with a starting cell density of about 3×10^5^ cells ml^−1^. Doxycycline was added to a final concentration of 300 pg ml^−1^ (minimum effective concentration) and incubated for 12 hours in order to induce *VSG221* RNAi. Cells were centrifuged, washed with 20 ml HMI-9, and then resuspended in HMI-9 with the appropriate drugs but without doxycycline. Total RNA was isolated at different time points and analysed using Northern blots as described above.

Recovery efficiency of the cells that resume growth after removal of doxycycline was determined by plating out serial dilutions (10x, 100x, 1000x and 10,000x) of the culture (at about 3×10^5^ cells ml^−1^) in fresh HMI-9 medium with appropriate drugs but without doxycycline. Each dilution was aliquotted over 48 wells of 96-well plates. The cloning efficiency using this method was also established using the parental *T. brucei* 221VB1.1 cell line in the absence of induction of *VSG221* RNAi.

In order to determine the cell volume, the parental *T. brucei* 221VB1.1 cell line and the *T. brucei* double expressers 221VP117.1 and 221VP117.2 were cultured to a concentration of 8–10×10^5^ cells ml^−1^. Fifty-fold dilutions of each culture were prepared in CASY® ton dilution liquid and the cell sizes were determined by using the Coulter size exclusion method on a CASY® Cell Counter and Analyser System model TT (Schärfe, Reutlingen, Germany) according to the manufacturer's instructions. The cell volumes were determined as an average of the measurements from five independent experiments.

### Protein analysis and *in vivo* metabolic labeling

For Western blot analysis, *T. brucei* 221VP117 cells were cultured and *VSG221* RNAi was induced by incubating the cultures with 1 µg ml^−1^ tetracycline for up to 96 hours. Cells were maintained at a concentration of about 7–10×10^5^ cells ml^−1^ throughout the experiment. Western blot analysis was performed by analysing lysate from 4×10^5^ cells per lane using 10% polyacrylamide gels, and transferred to nitrocellulose membrane using standard protocols. Blots were probed with antibodies raised against the N-termini of VSG117 or VSG221 (gift of Piet Borst) or BiP (gift of Jay Bangs). Anti-EP procyclin antibody is a mouse monoclonal antibody obtained from Cedarlane Laboratories (CLP0001A). Blots were imaged using Western Lightning® Detection Kit (Perkin Elmer) and a BioRad Fluor-S MAX imager.

For the metabolic labeling experiments, mid-logarithmic growth *T. brucei* 221VG1.1 cells induced with tetracycline (1 µg ml^−1^) for different times were centrifuged (800 g 10 minutes) and washed in minimal essential media minus either methionine or serine, before resuspension in the same media at 1×10^7^ cells ml^−1^. Cells were labeled for 1 hour at 37°C with either 5 µCi ml^−1^ [^35^S]-methionine (MP Biomedicals, 1175 Ci mmol^−1^) or 50 µCi ml^−1^ of [^3^H]-serine (20 Ci mmol^−1^, ARC) in a shaking water bath. The cells were collected by centrifugation and washed briefly in TDB buffer (25 mM KCl, 400 mM NaCl, 5 mM MgSO_4_, 100 mM Na_2_HPO_4_, NaH_2_PO_4_, 100 mM glucose) prior to samples taken for either protein or lipid analysis as previously described [26]. Proteins were separated on a 10% SDS-PAGE gel (4×10^6^ cell equivalents per lane) and visualised by Coomassie blue staining. To detect radiolabeled proteins the destained gel was soaked in En^3^hance™ (NEN) for 30 minutes, washed with water twice, soaked in 10% glycerol and dried and exposed to XAR-5 film at −70°C.

For the *in vivo* radiolabel incorporation into total, protein or lipid fractions *T. brucei* 221VG1.1 cells were induced with tetracycline (1 µg ml^−1^) for different times, and then labeled with [^35^S]-methionine or [^3^H]-serine for one hour as described above. Cells were split into three equal volumes and processed as follows: for total incorporation, cells were spun down through tetrachlorophenyl-modified silicone oil (Medford) and washed with TDB, prior to lysis with 1% SDS. Total uptake of radiolabel was quantified by scintillation counting. For incorporation into protein, cells were centrifuged and washed with TDB, followed by TCA protein precipitation and washing of the protein pellet, followed by reconstitution with 1% SDS and total radiolabel incorporation into protein quantified by scintillation counting. For the measurement of incorporation into lipids, cells were spun down and washed with TDB followed by extraction of lipids/glycolipids with CHCl_3_∶MeOH and CHCl_3_∶MeOH∶H_2_O (10∶10∶3). These extracts were desalted and the incorporation of radiolabel into the lipid fraction was quantified by scintillation counting. Values are means with standard deviations of three separate labeling experiments. The values at time zero are normalised to 100% whereby the deviation of total incorporation between different experiments was no greater than 10%.

### Amino acid analysis

For the amino acid analysis, triple aliquots of noninduced (30 ml of cells at a density of 1.5×10^6^) or cells where *VSG* RNAi had been induced with tetracycline for 24 hours (30 ml of cells at a density of 1.8×10^6^) were collected by centrifugation (850 g for 5 minutes at room temperature), washed twice briefly in ice-cold MEM (1 ml) and suspended in ice-cold MEM (250 µl, containing norleucine [2 µl of 1.5 mM] as internal standard). This cell suspension was immediately added to 1 ml 50°C ethanol to lyse the cells and cooled on ice for 10 minutes. After centrifugation at 14,000 rpm at 4°C for 30 minutes the supernatant was transferred to a new Eppendorf tube and dried under vacuum. An aliquot (10 µl) of a freshly prepared mixture (ethanol: sodium acetate [1 M]: triethylamine, [2∶2∶1 v/v]) was added, vortexed and thoroughly dried under vacuum. A 20 µl aliquot of a freshly prepared derivatising agent (water: triethylamine: and PITC phenylisothiocyanate [2∶2∶1 v/v]) was added to the dry samples, vortexed, centrifuged at full speed for one minute and allowed to stand at room temperature for 20 minutes, prior to drying under vacuum. The derivatised amino acids were dissolved in 100 µl of starting buffer and analysed using a PICO-TAG HPLC system (Waters). Triplicate samples of both a standard mix of amino acids containing 2 µl of 0.1–1.5 mM of each amino acid and an equivalent volume (250 µl) of MEM was used in order to correct the values obtained from the cell samples to obtain intracellular amino acid pools.

## Supporting Information

Figure S1 The cell volume of T. brucei expressing only VSG221 on its surface is not significantly different to that of T. brucei “double-expressors” expressing both VSG117 and VSG221 on their surface. The cell volumes were determined as pseudo-spheres using a CASY® Cell Counter. The parental T. brucei VB1.1 cell line is compared with that of the double-expressers T. brucei 221VP117.1 and 221VP117.2. A) The graph at the top shows data from a representative experiment whereby the percentage of cells with a respective cell diameter as pseudo-spheres is indicated. B) The values shown are the average of five independent experiments with the standard deviation indicated. The average peak volume is calculated from the projected cell diameter if the cells are represented as pseudo-spheres. C) The values shown below are the average of five independent experiments with the standard deviation indicated with error bars.
(0.24 MB TIF)

Figure S2 Global translation arrest after the induction of VSG RNAi monitored using [3H]-serine labeling of cells. A) T. brucei 221VG1.1 cells had VSG221 RNAi induced with tetracycline for the time in hours (h) indicated above, prior to labeling with [3H]-serine for 1 hour. Proteins were separated by SDS-PAGE and visualised by Coomassie staining (Coom.). B) Triplicate aliquots of the [3H]-serine labeled cells were processed to determine the mean rate of [3H]-serine incorporation into total protein versus time. The standard deviation is indicated with error bars.
(1.06 MB TIF)

Figure S3 Induction of a lethal RNAi mediated phenotype does not always trigger a global translation arrest in T. brucei. A) Various T. brucei cell lines with RNAi constructs allowing the tetracycline inducible knock-down of clathrin, PFR2, tubulin, actin or VSG221 were analysed. The levels of total protein synthesis were investigated after the induction of RNAi with tetracycline for 0 or 24 hours prior to labeling with [35S]-methionine for one hour. After this period cells were fully arrested. Total protein was separated on an SDS-PAGE gel. The top panel shows [35S]-labeled proteins detected by fluorography. Bottom panel is the corresponding Coomassie stained gel. B) Quantitation of triplicate samples of the of [35S]-methionine labeled cells after the induction of RNAi for 0 or 24 hours were processed to determine the mean rate (+/− the standard deviation) of [35S]-methionine incorporation into total protein after induction of the indicated knock-down.
(0.87 MB TIF)

Figure S4 Quantitation of the radioactive signal from the Northern blot analysis of transcripts from T. brucei 221VG1.1 cells where VSG221 RNAi has been induced for the respective time in hours (Fig. 3A). The signal is indicated as arbitrary units of radioactivity after quantitation was performed using a BioRad PhosphorImager and Quantity One software.
(0.18 MB TIF)

Figure S5 Blocking VSG synthesis by the induction of VSG RNAi does not result in downregulation of SL RNA, indicating that there is no induction of an SL response. RNA analysed was isolated from trypanosomes where VSG RNAi had been induced with tetracycline (Tet) for the time in hours (h) indicated above. Primer extension reactions to detect either the spliced leader (SL) RNA or the control U3 RNA were performed using radiolabeled oligonucleotides as described in the Experimental Procedures. The reaction products were electrophoresed on a polyacrylamide gel. The U3 or SL RNAs are indicated on the right.
(0.93 MB TIF)

Figure S6 A) 2D-DIGE comparison of cells grown in the presence or absence of VSG221 RNAi. T. brucei 221VB1.1 cells were either grown in the absence of tetracycline, or in the presence of tetracycline (Tet) for 8 hours to induce VSG221 RNAi. Three replicate sample pairs consisting of lysates from induced or noninduced cells were compared by 2D-DIGE. 2D-DIGE is a proteomic technique that allows the direct comparison of two sample types on a single gel. Each sample to be compared is pre-labeled with one of three CyDye DIGE fluor dyes (GE Healthcare). Here, one sample from each pair was labeled with CyDye5 and the other sample with CyDye3, such that in two of the pairs the noninduced sample was labeled with CyDye5 while in the other pair the noninduced sample was labeled with CyDye3. A standard sample consisting of an equal proportion of each of the six samples was generated and labeled with CyDye2. For each of the three replicates, the induced and noninduced samples, together with one third of the standard sample were combined and subjected to two dimensional electrophoresis. The standard sample is therefore present on all gels, and allows normalisation of protein abundance within each gel and statistical analysis across all gels. Proteins were separated in the first dimension on pH 3–11 NL IPG strips (GE Healthcare) and in the second dimension by SDS-PAGE-10% acrylamide: bisacrylamide 37.5∶1. On the 2D gels shown, molecular weight (Mw) decreases from top to bottom, and pH increases from left to right. Spots were visualised on a Typhoon scanner (GE Healthcare) and gel images were analysed and matched by reference to the standard sample using the DeCyder software suite (GE Healthcare). A total of 1486 spots were matched between at least two of the replicate gels and average ratios between induced and noninduced time points were obtained. B) No significant changes in protein levels were observed after the induction of VSG221 RNAi for 8 hours. Log10 average ratios of spots from induced and noninduced lysates were calculated for each of the 1486 spots analysed. The frequency of log10 ratios was calculated with a resolution of 0.025 and plotted as a percentage of the total number of spots analysed. Over 95% of the proteins showed a less than 25% change after the induction of VSG221 RNAi (area between the grey vertical bars), and no changes greater than 37% were observed.
(1.48 MB TIF)

Figure S7 There is no evidence for upregulation of procyclin after the induction of a VSG221 RNAi induced cell-cycle arrest in T. brucei VB1.1. A) Northern blot analysis does not show evidence for significant upregulation of procyclin after the induction of a VSG221 RNAi mediated cell-cycle arrest. RNA from the procyclic T. brucei 29-13 cell line (PF) was compared with RNA from the T. brucei 221VB1.1 cell line in which VSG221 RNAi had been induced with tetracycline for the time in hours (h) indicated above. The blot was hybridised with a probe for procyclin (CPT4) from [57], VSG221 to show the degree of VSG221 transcript knock-down, or actin as a loading control. Quantitation of the radioactive signal from the Northern blot analysis is indicated in arbitrary units of radioactivity, and was performed using a BioRad PhosphorImager with QuantityOne software. B) Western blot analysis of T. brucei VB1.1 stalled by the induction of VSG221 RNAi does not show evidence for the upregulation of EP procyclin. Protein lysates from bloodstream form T. brucei HNI(V02) [58] (BF), procyclic T. brucei 29-13 cell line (PF), or T. brucei 221VB1.1 where VSG221 RNAi had been induced for the time in hours (h) indicated above. The blot was probed with an antibody for EP procyclin or BiP as a loading control.
(0.32 MB TIF)

Figure S8 Titration of the minimum concentration of tetracycline or doxycycline which induces a maximal VSG221 RNAi mediated cell-cycle arrest. The T. brucei 221VB1.1 cell line was incubated with the indicated amount of tetracycline (Tet) or doxycycline (Dox) for the time in hours indicated below. The density of trypanosomes is indicated per ml x 105. The average of triplicate counts is shown, with the standard deviation indicated with error bars.
(0.28 MB TIF)

Figure S9 A) Cloning of recovered T. brucei 221VB1.1 cells after induction of VSG221 RNAi. Cells were induced using doxycycline for 12 hours, and then subsequently washed to remove the doxycycline. Ten-fold serial dilutions of washed cells were made and plated out over 48 wells of a 96 well plate with the indicated number of cells per well. The percentage of positive wells is indicated. Results are the average of three independent experiments with the standard deviation indicated with error bars. B) Cloning the parental T. brucei 221VB1.1 cells without the induction of VSG221 RNAi. Each serial dilution of the washed cells was plated out in 48 wells of a 96 well plate with the indicated number of cells per well. The percentage of positive wells is indicated. Results are the average of two independent experiments.
(0.19 MB TIF)
