# Supplementary figures and images for: Blocking Variant Surface Glycoprotein Synthesis in Trypanosoma brucei Triggers a General Arrest in Translation Initiation
Source: PLoS One. 2009 Oct 26;4(10):e7532. doi: 10.1371/journal.pone.0007532 (PMC2762041; doi:10.1371/journal.pone.0007532)

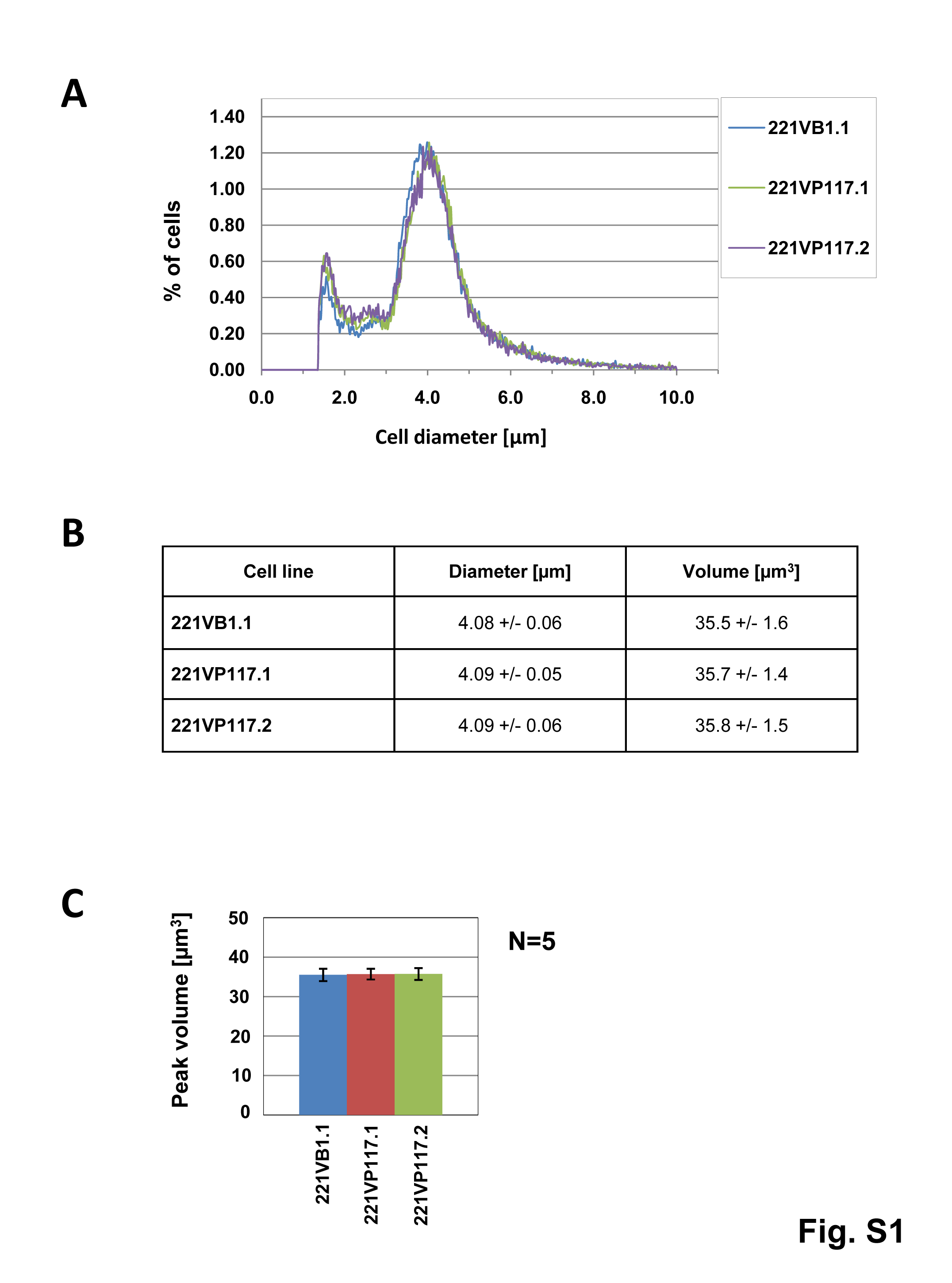

Supplement: Figure S1 — The cell volume of T. brucei expressing only VSG221 on its surface is not significantly different to that of T. brucei “double-expressors” expressing both VSG117 and VSG221 on their surface. The cell volumes were determined as pseudo-spheres using a CASY® Cell Counter. The parental T. brucei VB1.1 cell line is compared with that of the double-expressers T. brucei 221VP117.1 and 221VP117.2. A) The graph at the top shows data from a representative experiment whereby the percentage of cells with a respective cell diameter as pseudo-spheres is indicated. B) The values shown are the average of five independent experiments with the standard deviation indicated. The average peak volume is calculated from the projected cell diameter if the cells are represented as pseudo-spheres. C) The values shown below are the average of five independent experiments with the standard deviation indicated with error bars. (0.24 MB TIF) [file pone.0007532.s001.tif]

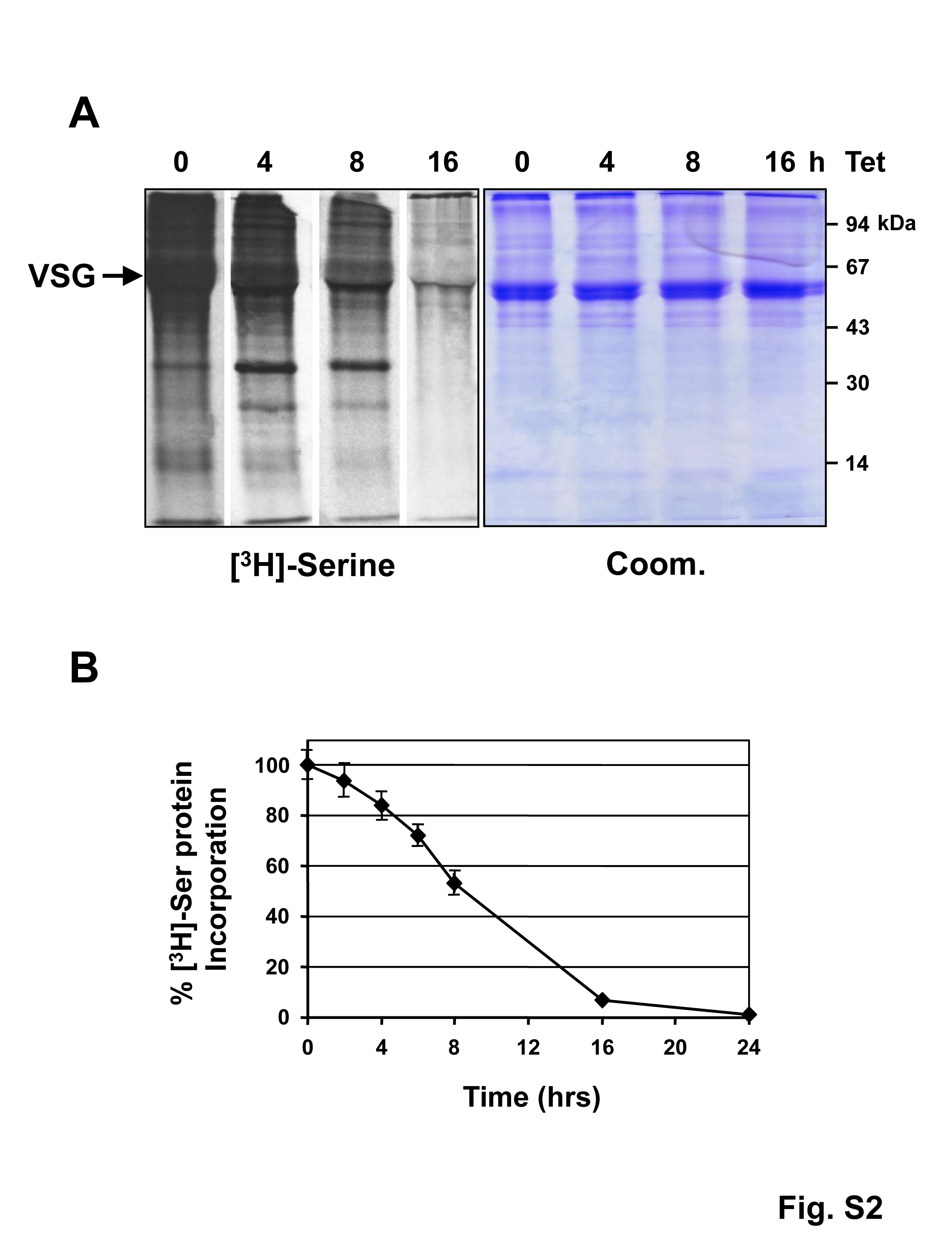

Supplement: Figure S2 — Global translation arrest after the induction of VSG RNAi monitored using [3H]-serine labeling of cells. A) T. brucei 221VG1.1 cells had VSG221 RNAi induced with tetracycline for the time in hours (h) indicated above, prior to labeling with [3H]-serine for 1 hour. Proteins were separated by SDS-PAGE and visualised by Coomassie staining (Coom.). B) Triplicate aliquots of the [3H]-serine labeled cells were processed to determine the mean rate of [3H]-serine incorporation into total protein versus time. The standard deviation is indicated with error bars. (1.06 MB TIF) [file pone.0007532.s002.tif]

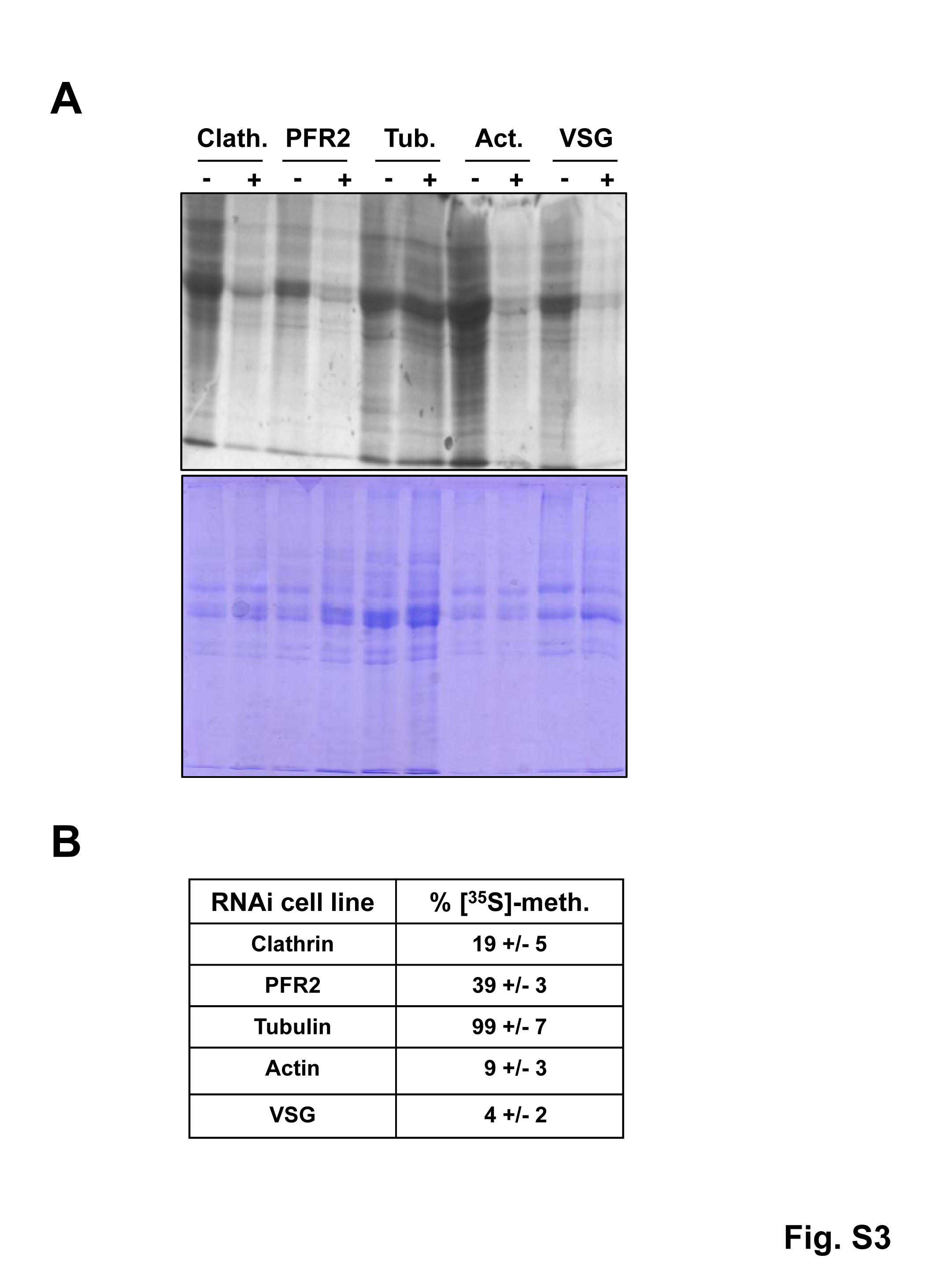

Supplement: Figure S3 — Induction of a lethal RNAi mediated phenotype does not always trigger a global translation arrest in T. brucei. A) Various T. brucei cell lines with RNAi constructs allowing the tetracycline inducible knock-down of clathrin, PFR2, tubulin, actin or VSG221 were analysed. The levels of total protein synthesis were investigated after the induction of RNAi with tetracycline for 0 or 24 hours prior to labeling with [35S]-methionine for one hour. After this period cells were fully arrested. Total protein was separated on an SDS-PAGE gel. The top panel shows [35S]-labeled proteins detected by fluorography. Bottom panel is the corresponding Coomassie stained gel. B) Quantitation of triplicate samples of the of [35S]-methionine labeled cells after the induction of RNAi for 0 or 24 hours were processed to determine the mean rate (+/− the standard deviation) of [35S]-methionine incorporation into total protein after induction of the indicated knock-down. (0.87 MB TIF) [file pone.0007532.s003.tif]

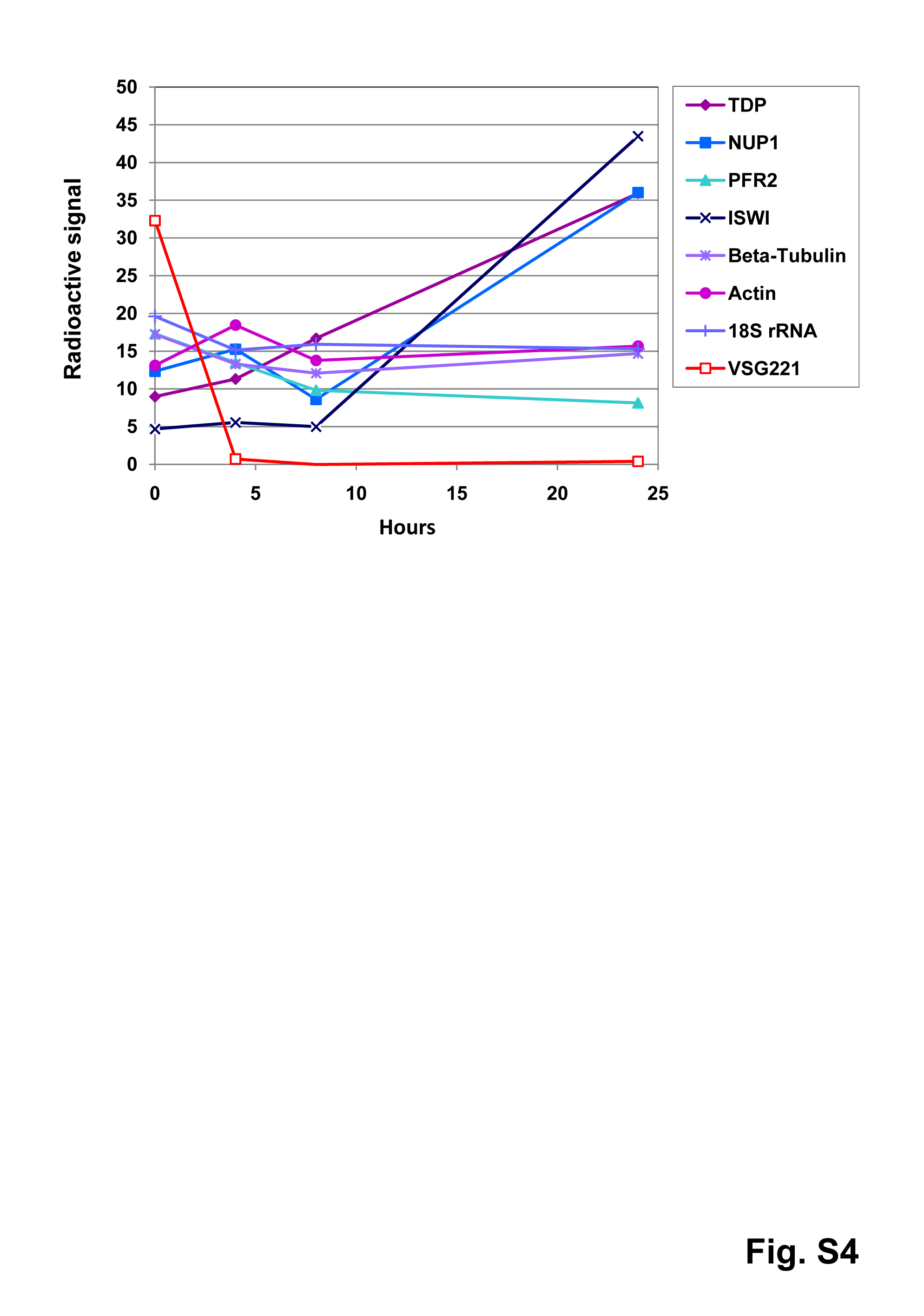

Supplement: Figure S4 — Quantitation of the radioactive signal from the Northern blot analysis of transcripts from T. brucei 221VG1.1 cells where VSG221 RNAi has been induced for the respective time in hours (Fig. 3A). The signal is indicated as arbitrary units of radioactivity after quantitation was performed using a BioRad PhosphorImager and Quantity One software. (0.18 MB TIF) [file pone.0007532.s004.tif]

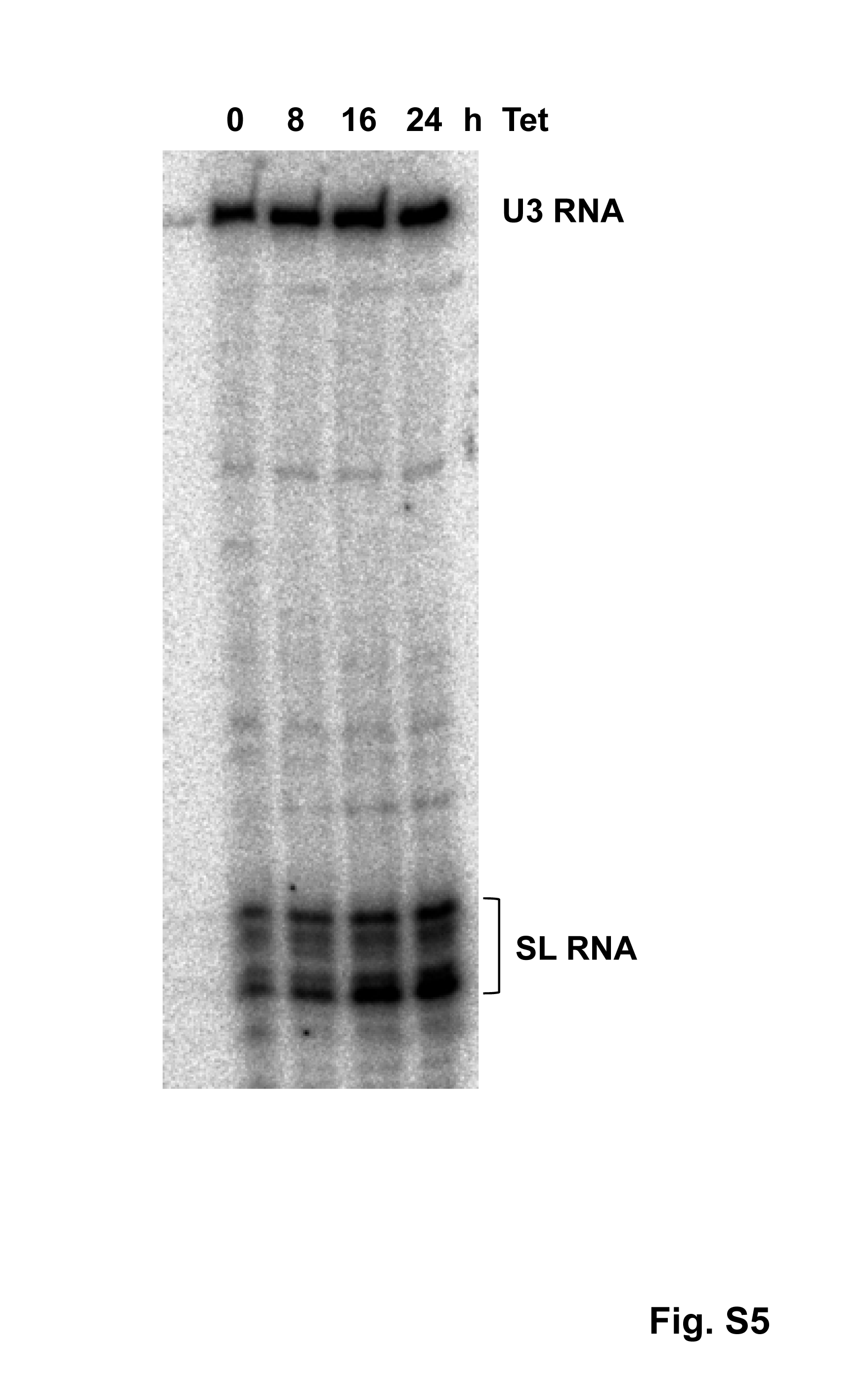

Supplement: Figure S5 — Blocking VSG synthesis by the induction of VSG RNAi does not result in downregulation of SL RNA, indicating that there is no induction of an SL response. RNA analysed was isolated from trypanosomes where VSG RNAi had been induced with tetracycline (Tet) for the time in hours (h) indicated above. Primer extension reactions to detect either the spliced leader (SL) RNA or the control U3 RNA were performed using radiolabeled oligonucleotides as described in the Experimental Procedures. The reaction products were electrophoresed on a polyacrylamide gel. The U3 or SL RNAs are indicated on the right. (0.93 MB TIF) [file pone.0007532.s005.tif]

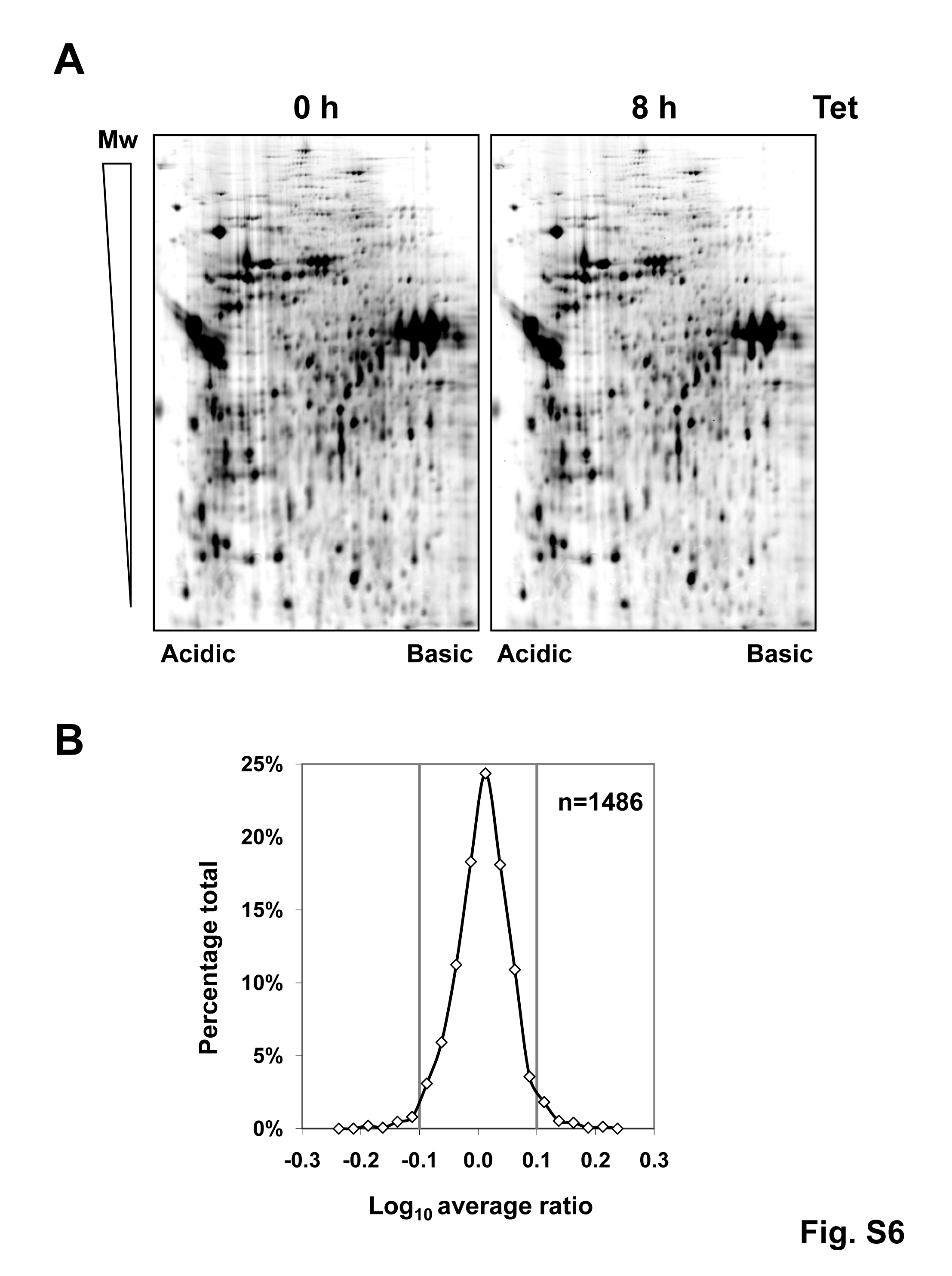

Supplement: Figure S6 — A) 2D-DIGE comparison of cells grown in the presence or absence of VSG221 RNAi. T. brucei 221VB1.1 cells were either grown in the absence of tetracycline, or in the presence of tetracycline (Tet) for 8 hours to induce VSG221 RNAi. Three replicate sample pairs consisting of lysates from induced or noninduced cells were compared by 2D-DIGE. 2D-DIGE is a proteomic technique that allows the direct comparison of two sample types on a single gel. Each sample to be compared is pre-labeled with one of three CyDye DIGE fluor dyes (GE Healthcare). Here, one sample from each pair was labeled with CyDye5 and the other sample with CyDye3, such that in two of the pairs the noninduced sample was labeled with CyDye5 while in the other pair the noninduced sample was labeled with CyDye3. A standard sample consisting of an equal proportion of each of the six samples was generated and labeled with CyDye2. For each of the three replicates, the induced and noninduced samples, together with one third of the standard sample were combined and subjected to two dimensional electrophoresis. The standard sample is therefore present on all gels, and allows normalisation of protein abundance within each gel and statistical analysis across all gels. Proteins were separated in the first dimension on pH 3–11 NL IPG strips (GE Healthcare) and in the second dimension by SDS-PAGE-10% acrylamide: bisacrylamide 37.5∶1. On the 2D gels shown, molecular weight (Mw) decreases from top to bottom, and pH increases from left to right. Spots were visualised on a Typhoon scanner (GE Healthcare) and gel images were analysed and matched by reference to the standard sample using the DeCyder software suite (GE Healthcare). A total of 1486 spots were matched between at least two of the replicate gels and average ratios between induced and noninduced time points were obtained. B) No significant changes in protein levels were observed after the induction of VSG221 RNAi for 8 hours. Log10 average ratios of spots from ind [file pone.0007532.s006.tif]

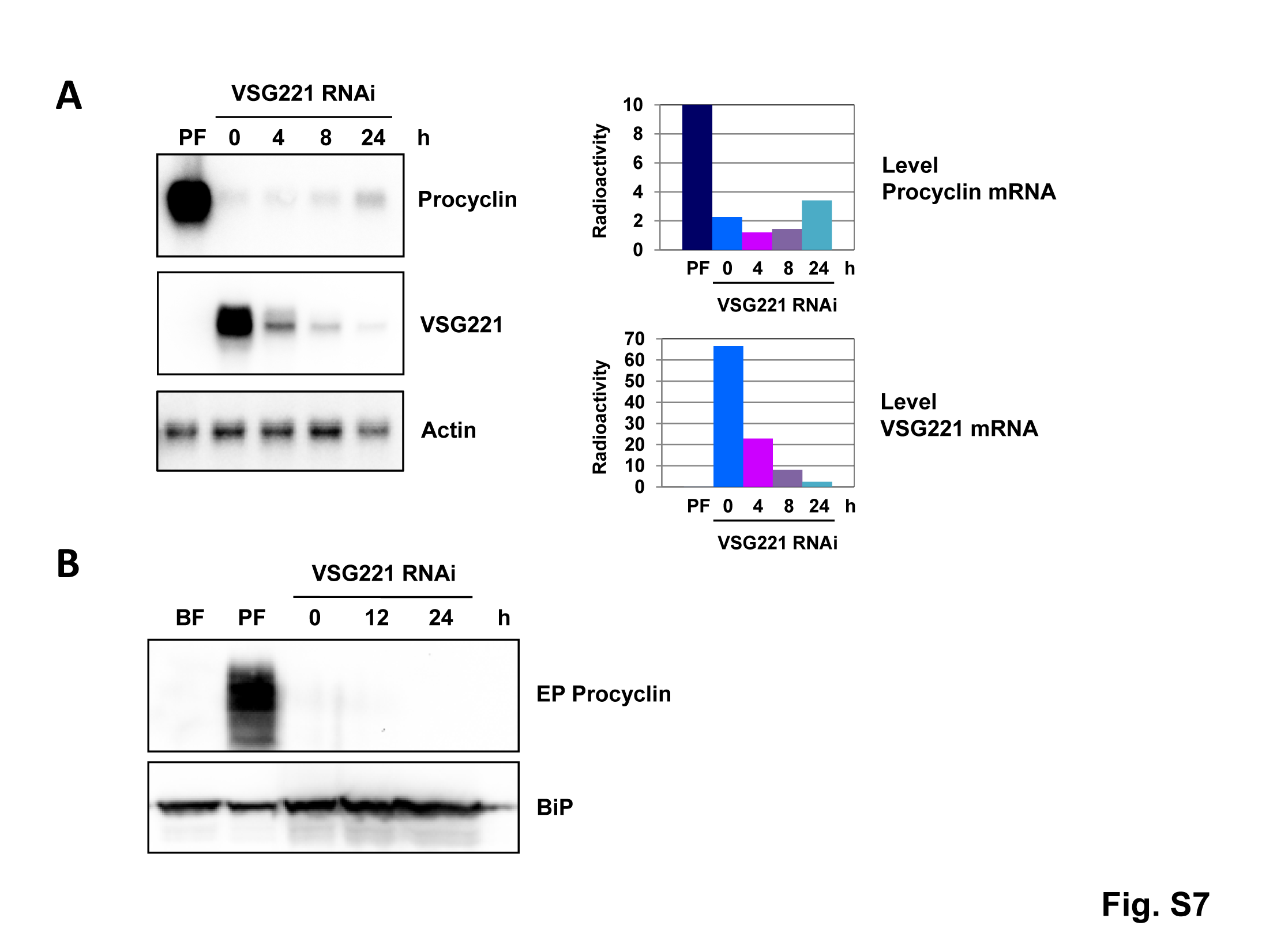

Supplement: Figure S7 — There is no evidence for upregulation of procyclin after the induction of a VSG221 RNAi induced cell-cycle arrest in T. brucei VB1.1. A) Northern blot analysis does not show evidence for significant upregulation of procyclin after the induction of a VSG221 RNAi mediated cell-cycle arrest. RNA from the procyclic T. brucei 29-13 cell line (PF) was compared with RNA from the T. brucei 221VB1.1 cell line in which VSG221 RNAi had been induced with tetracycline for the time in hours (h) indicated above. The blot was hybridised with a probe for procyclin (CPT4) from [57], VSG221 to show the degree of VSG221 transcript knock-down, or actin as a loading control. Quantitation of the radioactive signal from the Northern blot analysis is indicated in arbitrary units of radioactivity, and was performed using a BioRad PhosphorImager with QuantityOne software. B) Western blot analysis of T. brucei VB1.1 stalled by the induction of VSG221 RNAi does not show evidence for the upregulation of EP procyclin. Protein lysates from bloodstream form T. brucei HNI(V02) [58] (BF), procyclic T. brucei 29-13 cell line (PF), or T. brucei 221VB1.1 where VSG221 RNAi had been induced for the time in hours (h) indicated above. The blot was probed with an antibody for EP procyclin or BiP as a loading control. (0.32 MB TIF) [file pone.0007532.s007.tif]

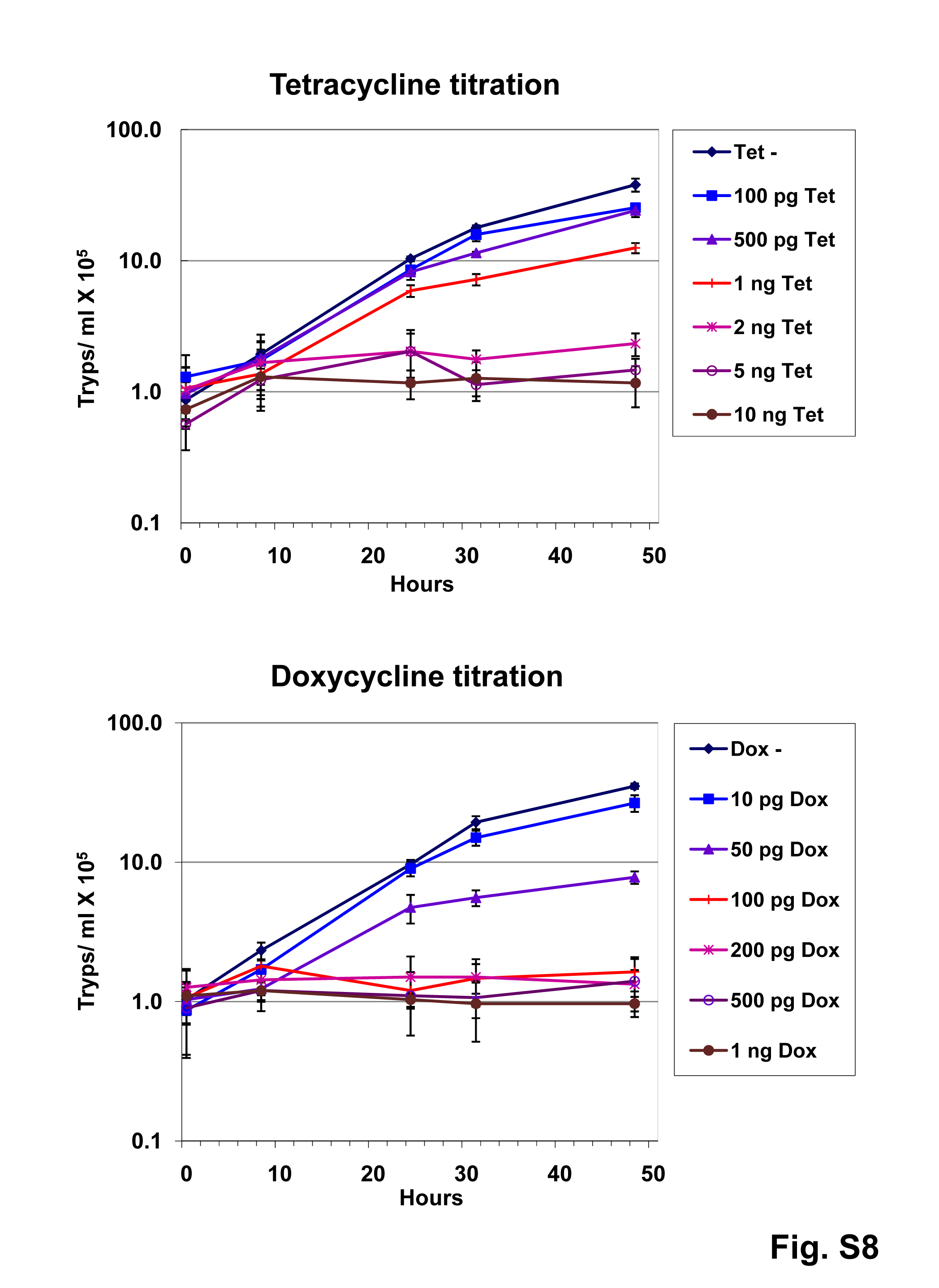

Supplement: Figure S8 — Titration of the minimum concentration of tetracycline or doxycycline which induces a maximal VSG221 RNAi mediated cell-cycle arrest. The T. brucei 221VB1.1 cell line was incubated with the indicated amount of tetracycline (Tet) or doxycycline (Dox) for the time in hours indicated below. The density of trypanosomes is indicated per ml x 105. The average of triplicate counts is shown, with the standard deviation indicated with error bars. (0.28 MB TIF) [file pone.0007532.s008.tif]

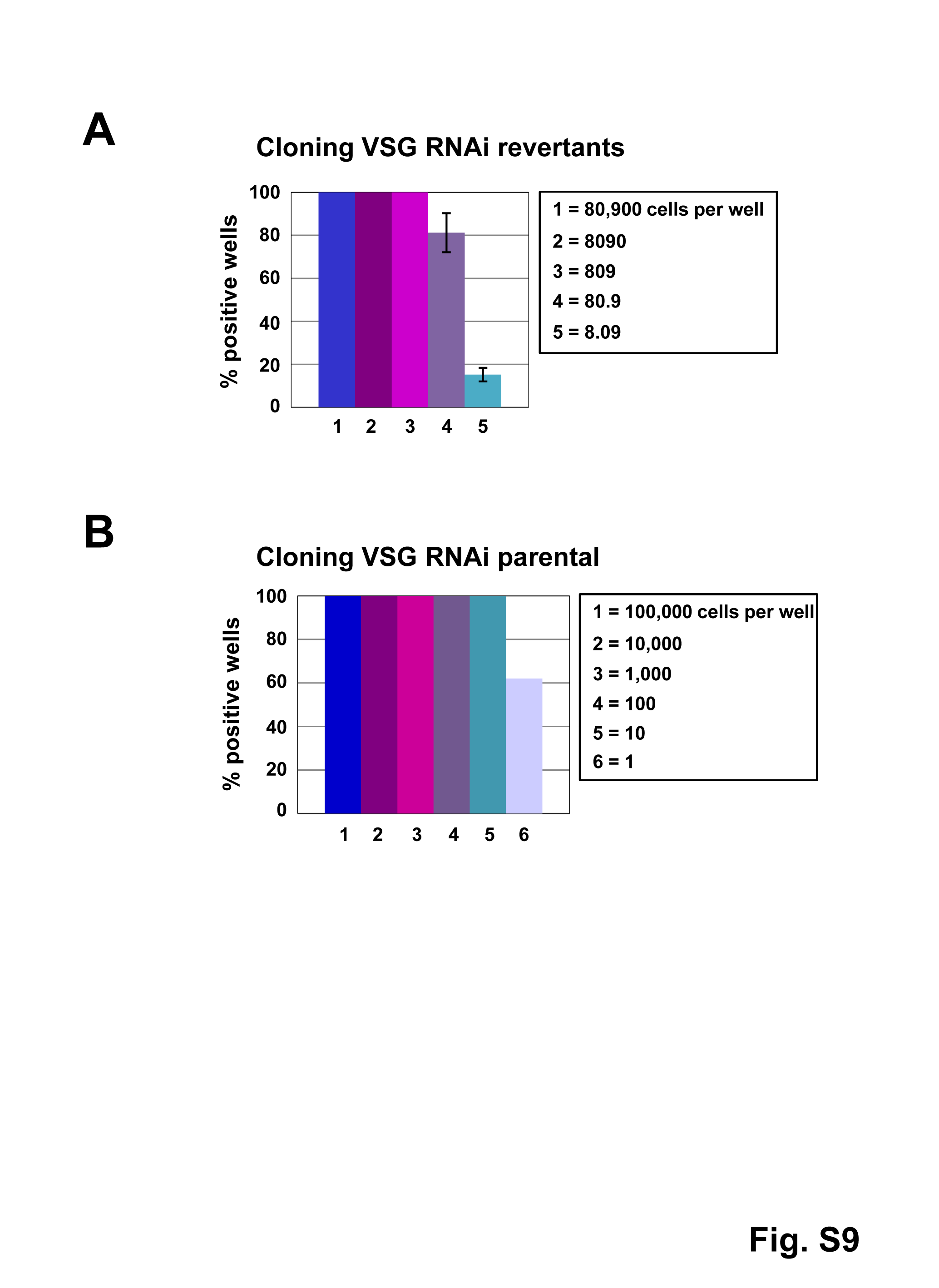

Supplement: Figure S9 — A) Cloning of recovered T. brucei 221VB1.1 cells after induction of VSG221 RNAi. Cells were induced using doxycycline for 12 hours, and then subsequently washed to remove the doxycycline. Ten-fold serial dilutions of washed cells were made and plated out over 48 wells of a 96 well plate with the indicated number of cells per well. The percentage of positive wells is indicated. Results are the average of three independent experiments with the standard deviation indicated with error bars. B) Cloning the parental T. brucei 221VB1.1 cells without the induction of VSG221 RNAi. Each serial dilution of the washed cells was plated out in 48 wells of a 96 well plate with the indicated number of cells per well. The percentage of positive wells is indicated. Results are the average of two independent experiments. (0.19 MB TIF) [file pone.0007532.s009.tif]
